# Integrating Senescence and Oxidative Stress in Cardiac Disease

**DOI:** 10.3390/ijms262411917

**Published:** 2025-12-10

**Authors:** Hyeong Rok Yun, Manish Kumar Singh, Sunhee Han, Jyotsna S. Ranbhise, Joohun Ha, Sung Soo Kim, Insug Kang

**Affiliations:** 1 Department of Biochemistry and Molecular Biology, School of Medicine, Kyung Hee University, Seoul 02447, Republic of Korea; foryou018@naver.com (H.R.Y.);; 2 Biomedical Science Institute, Kyung Hee University, Seoul 02447, Republic of Korea; 3 Department of Biomedical Science, Graduate School, Kyung Hee University, Seoul 02447, Republic of Korea

**Keywords:** oxidative stress, cellular senescence, cardiac disease

## Abstract

Cellular senescence and oxidative stress constitute an interdependent axis that underlies cardiac pathophysiology. Cellular senescence, defined as durable proliferative arrest, is initiated and sustained by redox imbalance, whereas mitochondrial reactive oxygen species function as signaling molecules or mediators of injury. In the heart, cellular senescence and oxidative stress influence remodeling and dysfunction across diseases, including ischemia–reperfusion injury, heart failure with preserved ejection fraction, dilated cardiomyopathy, and cardiac hypertrophy. Accordingly, delineating stress adaptation in cellular senescence is essential for elucidating oxidative stress-related pathogenesis. In this review, we attempt to provide an overview of the fundamental mechanisms and functions of cellular senescence in response to oxidative stress and redox signaling in disease. In addition, we integrate experimental and clinical evidence and delineate implications for mechanism-informed prevention and therapy.

## 1. Introduction

Cellular senescence denotes a durable state of proliferative arrest accompanied by sustained viability and active metabolism [1,2]. First described in 1961 in normal human fibroblasts, senescent cells elucidate a characteristic secretory program [3], the senescence-associated secretory phenotype (SASP), which comprises cytokines, chemokines, growth factors, matrix-remodeling enzymes, and other mediators that act via autocrine and paracrine signaling [4,5]. The biological consequences of cellular senescence are largely determined by cell type, the tissue microenvironment surroundings, and the duration of senescent-cell persistence. In the heart, sustained oxidative stress induces cellular senescence and SASP in endothelial cells, cardiomyocytes, and fibroblasts, which in turn aggravates oxidative injury and inflammation, ultimately leading to vascular dysfunction, maladaptive cardiac remodeling, and the progression of cardiac diseases [6,7,8]. Senescence is induced by multiple upstream stressors, including telomere shortening, oncogenic activation, mitochondrial dysfunction, and maladaptive redox signaling [2,9,10,11]. Among these factors, oxidative stress is predominant driver. Reactive oxygen species (ROS) damage DNA, proteins, and lipids and are thus recognized as contributors to senescence [12,13]. Sustained ROS alters adaptive signaling toward chronic tissue injury [14]. 

In the heart, cellular senescence represents a key pathobiological mechanism [15,16]. Cellular senescence is accompanied by DNA damage and mitochondrial dysfunction, which drive the development and progression of cardiac disease [17,18]. This review synthesizes current concepts and mechanisms of senescence and oxidative stress in cardiac diseases, outlines their manifestations across major clinical phenotypes, and directly informs the development of preventive and therapeutic strategies. 

## 2. Biological Pathway of Senescence and Oxidative Stress

### 2.1. Mechanisms of the Senescence Pathway

Cellular senescence is a stable program of growth arrest accompanied by a persistent SASP. Senescence is a conserved cellular response to DNA damage [19], telomere dysfunction [20], oncogenic activation [21], proteostasis collapse [22], and oxidative stress [23]. In response to these cellular stresses in diverse tissues, the canonical DNA damage response (DDR) activates ataxia-telangiectasia-mutated (ATM) and ATM- and Rad3-related (ATR) proteins, which phosphorylate checkpoint kinases 1 and 2 (CHK1/CHK2) and promote tumor protein P53 (p53) stabilization with the induction of cyclin-dependent kinase inhibitor 1A (p21/CDKN1A) [24,25,26]. In addition, stress- and chromatin-mediated pathways promote cyclin-dependent kinase inhibitor 2A (p16/CDKN2A) and the retinoblastoma protein (RB)-dependent suppression of E2 promoter-binding factor (E2F)-driven transcription [27,28]. At the cell-cycle checkpoint levels, the p53/p21 and p16/RB enhance cell-cycle arrest through inhibition of cyclin-dependent kinases 2, 4, and 6 (CDK2/4/6), maintaining RB in a hypophosphorylated state and preventing transition to S-phase [29,30,31]. The predominance of p16/RB is associated with stable G1 arrest, whereas p53/p21 supports G1 or G2 arrest, depending on stress type, intensity, and cell state [32,33,34]. Downstream of CDK suppression, the dimerization partner (DP), RB-like (RBL1/p107, RBL2/p130), E2F, and MuvB (DREAM) complex assembles to silence a broad mitotic gene program and acts as a key effector of arrest, independent of canonical E2F regulation [35,36,37]. RB-linked circuitry further cooperates with anaphase-promoting complex/cyclosome (APC/C) and APC/C co-activator CDC20 homolog 1 (CDH1) to restrain mitotic cyclins, establishing a second layer that prevents cell-cycle re-entry [38,39,40,41]. The balance between the p53/p21 and the p16/RB axes modulates cell-fate decisions toward apoptosis, adaptive quiescence, or a stable senescent state. Resistance to apoptosis is a characteristic of senescence. Apoptosis is maintained by B-cell lymphoma-2 (BCL-2) family prosurvival proteins and reprogrammed stress-response signaling [42,43,44]. Senescence is generally stable but not irreversible. Under specific conditions, including mammalian target of rapamycin complex 1 (mTORC1) inhibition with rapamycin [45], Janus kinase 1 and 2 (JAK1/2) blockade [46], bromodomain and extraterminal (BET)-bromodomain-containing protein 4 (BRD4) inhibition [47], p38-mitogen-activated protein kinase (MAPK) to MAPK-activated protein kinase 2 (MK2) pathway inhibition [27], or blockade of cyclic GMP-AMP synthase (cGAS)-stimulator of interferon gene (STING) pathway [48], secretory metabolites [49,50] are reduced without loss of cell-cycle arrest.

Telomere dysfunction is a canonical trigger of senescent arrest [20]. Replication-driven telomere shortening exposes chromosome ends and triggers persistent DNA-damage signaling at telomeric foci that resemble one-ended double-strand breaks [20,51]. Telomere-associated DNA damage foci are structurally stable lesions that evade efficient repair and sustain checkpoint signaling [52]. Senescent cells accumulate DNA segments with chromatin alterations, reinforcing senescence (DNA-SCARS) marked by γH2AX and 53BP1 [53]. DNA-SCARS exhibit minimal recruitment of late repair factors, indicating attenuated homologous recombination and increased reliance on end joining [52,53]. Replication stress and R-loop stabilization lead to replication-fork collapse and the formation of persistent DNA damage foci [52]. In addition to telomere dysfunction, oncogene activation triggers replication stress and nucleotide imbalance, which activate the DNA-damage response and establish senescent arrest [54]. Loss of tumor suppressor function promotes senescent arrest via dysregulated growth-factor signaling and metabolic reprogramming [55,56,57]. Phosphatidylinositol 3-kinases (PI3K)-protein kinase B (AKT)-mammalian target of rapamycin (mTOR) signaling promotes p53/p21-mediated senescent arrest, reflecting diverse upstream regulation [58]. Mitogenic hyperactivation elevates nucleotide demand and promotes transcription–replication conflicts, sustaining checkpoint signaling despite limited detectable double-stranded breaks [59,60,61].

SASP is initiated by nuclear factor-kappa B (NF-κB), p38 MAPK signaling, and CCAAT/enhancer-binding protein beta (C/EBPβ), subsequently requiring chromatin-dependent gene expression involving bromodomain-containing protein 4 (BRD4) and EP300 [47,62]. The core secretory module encompasses interleukin-6 (IL-6), interleukin-8 (IL-8), and monocyte chemoattractant protein-1 (MCP-1), alongside matrix metalloproteinases (MMPs) and associated regulatory factors [63,64,65]. However, the precise composition of the SASP is highly heterogeneous and depends on the cell type and the specific senescence-inducing stimulus. For instance, senescent fibroblasts tend to secrete extracellular matrix (ECM)-remodeling enzymes, whereas senescent cardiomyocytes often release specific growth factors and matricellular proteins. Cell enlargement and cytoskeletal remodeling facilitate the nuclear translocation of GATA4 and NF-κB. The autophagic control of GATA4 connects lysosome function to secretory intensity [50]. Translational and post-transcriptional control acts through mTORC1, the p38-to-MK2 pathway, and AU-rich element-binding proteins that determine messenger RNA (mRNA) stability [4,66]. Notch signaling mediates secondary senescence via juxtacrine signaling [49,67]. Early IL-1-primed SASP is distinct from a later chromatin-licensed SASP [66]. Cells shift between an inflammatory- and TGF-β-dominant state as conditions change [49]. MK2-dependent phosphorylation of RNA-binding proteins stabilizes AU-rich SASP transcripts, maintaining secretion despite upstream variability [68].

Large-scale epigenetic remodeling stabilizes senescence through heterochromatin redistribution and altered enhancer activity at inflammatory genes. Senescence-associated heterochromatin foci (SAHFs) are compact nuclear domains enriched in repressive chromatin marks, such as histone H3 lysine 9 trimethylation (H3K9me3), and architectural proteins, such as heterochromatin protein 1 (HP1) and macroH2A [69,70,71]. SAHF formation depends on cell type and trigger and is prominent in oncogene-induced senescence. Loss of nuclear lamina integrity promotes micronuclei formation and the release of chromatin fragments into the cytosol [72]. Reduced lamin-B1 (LMNB1), together with loss of high-mobility group box 2 (HMGB2), accompanies nuclear reorganization and reduces chromatin boundaries around inflammatory genes [73,74]. Cytosolic DNA activates cGAS-STING signaling. The resulting signaling induces type I interferon (IFN) and NF-κB signaling that sustains the secretory phenotype and promotes immune cell infiltration [75]. The reactivation of retroelements generates double-stranded RNA that activates innate RNA sensors such as retinoic acid inducible gene-I (RIG-I), melanoma differentiation-associated gene-5 (MDA-5), and Toll-like receptor-3 (TLR-3). The activation of these sensors enhances IFN and stabilizes SASP [76,77,78]. DNA methylation and chromatin accessibility change in partly conserved patterns quantified by DNA methylation clocks [79]. Together, these chromatin and nucleic acid-sensing pathways sustain the persistence of cellular senescence and its paracrine effect. 

**Figure 1:**
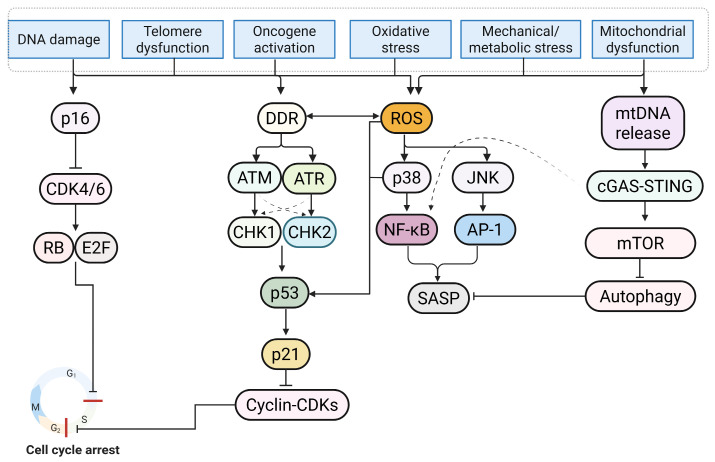
Senescence is triggered by diverse upstream stressors, including DNA damage, telomere dysfunction, oncogene activation, oxidative stress, and mitochondrial or metabolic dysfunction. These heterogeneous insults trigger the DNA damage response (DDR). Within this signaling cascade, ataxia-telangiectasia-mutated (ATM) and ATM- and Rad3-related (ATR) kinases display reciprocal regulation and phosphorylate checkpoint kinase 2 (CHK2) and checkpoint kinase 1 (CHK1), respectively, resulting in tumor protein p53 (p53) stabilization and cyclin-dependent kinase inhibitor 1A (p21) induction. Upon activation, p21 suppresses cyclin-dependent kinase (CDK) complexes, thereby establishing dual blockade at both the G1/S and G2/M checkpoints (indicated by red bars). Concomitantly, the cyclin-dependent kinase inhibitor 2A (p16) axis inhibits cyclin-dependent kinase 4 and 6 (CDK4/6), maintaining retinoblastoma protein (RB) in a hypophosphorylated state and silencing E2 promoter-binding factor (E2F)-dependent transcription to consolidate G1/S arrest. Independent of cell-cycle control, mitochondrial reactive oxygen species (ROS) and metabolic cues stimulate the p38 mitogen-activated protein kinase (p38 MAPK)/c-Jun N-terminal kinase (JNK) and mechanistic target of rapamycin (mTOR) pathways. mTOR drives senescence by suppressing autophagy, while p38 MAPK/JNK signaling activates nuclear factor-kappa B (NF-κB) and activator protein-1 (AP-1) to orchestrate the expression of the senescence-associated secretory phenotype (SASP). Furthermore, the cyclic GMP-AMP synthase (cGAS)-stimulator of interferon gene (STING) pathway, triggered by cytosolic mitochondrial DNA (mtDNA), exacerbates inflammatory signaling. In concert, these signaling nodes dictate stable growth arrest and the secretory phenotype (Figure created in Biorender. Hyeong Rok Yun. (2025) https://app.biorender.com/illustrations/canvas-beta/6911a5a2ecdb3e3f73bc83cf).

Organelle quality control provides a mechanistic basis for canonical markers of cellular senescence [80]. Lysosomal enlargement with increased senescence-associated beta-galactosidase activity (SA-β-gal) and lipofuscin accumulation is characteristic of senescence [81,82]. Autophagic flux is bidirectionally linked with cellular senescence [83]. Impairment of autophagy promotes the accumulation of damaged proteins and organelles, whereas induction of autophagy attenuates the SASP [84]. Endoplasmic reticulum (ER) stress and the integrated stress response modulate translation via the phosphorylation of eukaryotic initiation factor-2 alpha (eIF2α) and reprogram amino acid and lipid metabolism with a state of cell-cycle arrest [85,86]. There is no single definitive marker. Diagnostic marker combinations integrating SA-β-gal with p16 or p21, loss of LMNB1, telomere-associated damage foci, heterochromatin features, and SASP profiling are best implemented using a single-cell or spatial assay to reduce classification errors [2,87]. To circumvent the inherent limitations of invasive tissue sampling, contemporary research has pivoted toward non-invasive detection modalities to enhance clinical applicability. Liquid biopsy strategies, encompassing the quantification of senescence-associated extracellular vesicles (SA-EVs) and circulating analytes such as growth differentiation factor 15 (GDF15), constitute a scalable paradigm for longitudinal patient surveillance. Concurrently, advancements in molecular imaging, particularly positron emission tomography (PET) utilizing radiotracers specific to senescence-associated enzymes, facilitate the in vivo spatiotemporal quantification of cardiac senescence accumulation [88,89,90,91]. An overview of the senescence-associated cell-cycle arrest pathway is shown in Figure 1.

### 2.2. Molecular Biology of Oxidative Stress

Reactive oxygen species (ROS) are oxygen (O_2_)-containing molecules with high chemical reactivity. Most cellular oxygen is fully reduced to water via mitochondrial electron transport (mETC), and only a small cellular oxygen is diverted to ROS through electron leakage [92]. During oxidative phosphorylation, complexes I and III generate superoxide (O_2_•^−^), which is converted by superoxide dismutase 1 and 2 (SOD1/2) into hydrogen peroxide (H_2_O_2_) [93,94]. In the presence of ferrous iron, H_2_O_2_ participates in Fenton chemistry, producing hydroxyl radical (HO•), an extremely reactive species that damages lipids, proteins, and nucleic acids [95]. Lipid peroxidation is a chain reaction that initiates as HO• removes a bis-allylic hydrogen from a polyunsaturated fatty acid (PUFA) to form a carbon-centered radical (R•) [96]. Molecular O_2_ produces a lipid peroxyl radical (ROO•), which propagates the reaction and gives rise to lipid hydroperoxides (ROOH) [97]. Glutathione peroxidase-4 (GPx4) reduces ROOH to the corresponding alcohol (ROH) using glutathione, thereby limiting membrane damage and cytotoxicity [98]. NADPH oxidase 2 (NOX2) serves as a major non-mitochondrial source of ROS in innate immune cells. Upon microbial stimulation, NOX2 transfers electrons from NADPH to O_2_, generating O_2_•^−^ within the phagosomal lumen or extracellular space [99]. SODs subsequently convert the resulting O_2_•^−^ flux into H_2_O_2_, at which point myeloperoxidase then reacts with chloride to generate hypochlorous acid (HOCl) [100]. Oxidants are essential for pathogen killing, but excessive production injures host tissues. Other enzymes, including DUOX1/2, xanthine oxidase, cytochrome P450s, monoamine oxidases, and nitric oxide synthases (NOSs), produce ROS or reactive nitrogen species (RNS) in distinct subcellular locations [101,102]. O_2_•^−^ reacts rapidly with nitric oxide (NO) to form peroxynitrite (ONOO^−^) [103]. To limit oxidative stress, cells rely on enzyme-based and small-molecule antioxidant defenses. Core enzymes include SOD, catalase, glutathione peroxidases (GPxs), glutathione reductase (GR), peroxiredoxins (Prxs), and the thioredoxin (Trx) system [104,105]. Non-enzymatic antioxidants include reduced glutathione (GSH), ascorbate, tocopherols, urate, and carotenoids, while metal-binding proteins such as transferrin, ferritin, ceruloplasmin, and albumin restrict iron and copper availability and thereby suppress Fenton reactions [105,106]. At the transcriptional level, Kelch-like ECH-associated protein 1 (Keap1)/nuclear factor erythroid 2-related factor 2 (NRF2) pathway is a major regulator of the antioxidant defense system. Under normal conditions, Keap1 with cullin 3 (Cul3) targets NRF2 for proteasomal degradation, whereas oxidative stress stabilizes NRF2, promotes nuclear translocation, and activates antioxidant response element programs that upregulate heme oxygenase 1 (HMOX1), NAD(P)H:quinone oxidoreductase 1 (NQO1), SOD and Gpxs, ferritin, Trx, and related detoxifying systems [107,108,109].

### 2.3. Interplay Between Cellular Senescence and Oxidative Stress

Oxidative stress has been implicated as a primary trigger of cellular senescence. Excess ROS leads to DNA damage and a sustained DDR, which activates p53/p21 and p16/RB [110,111]. In parallel, redox-sensitive transcription factors, such as NF-κB and C/EBPβ, are activated and amplify SASP [62,112,113]. However, it is not yet clear whether the antioxidant defense program represents a nonspecific response to increased oxidant or a ROS-dependent response. Nevertheless, cellular senescence is accompanied by the upregulation of antioxidant defense genes [18,114,115]. Moreover, mitochondria in the senescent cells typically exhibit decreased mitochondrial respiratory capacity and membrane potential (ΔΨm), accompanied by increased ROS production [113,116]. Excess dynamin-related protein 1 (DRP1) activity with decreased mitofusins 1 and 2 (MFN1/2) and optic atrophy protein (OPA1) leads to cristae destabilization, elevated ROS, and reduced respiratory capacity [117,118,119]. Senescence-associated mitochondrial dysfunction (SAMD) is characterized by primary organelle injury with oxidized nicotinamide adenine dinucleotide (NAD^+^) depletion and reduced sirtuin (SIRT) activity, accompanied by reprogrammed SASP [18,120,121]. SAMD can be driven by persistent DDR through pathways including p38 MAPK, TGF-β, and Nf-κB signaling, leading to mitochondrial fragmentation, loss of ΔΨm, and impaired complex I and II respiration [27,112,122]. In addition, the dysregulated opening of the mitochondrial permeability transition pore (mPTP) has been implicated as a key effector that links mitochondrial calcium (Ca^2^⁺) overload and oxidative stress to SAMD. In the cardiac system, maladaptive mPTP opening promotes loss of ΔΨm, ATP depletion, and cardiomyocyte death in models of ischemia–reperfusion injury and chronic remodeling, suggesting that mPTP activity may provide an opportunity to selectively target senescent cardiac cells [123,124,125,126].

Early mitochondrial injury triggers mitochondrial DNA (mtDNA) efflux into the cytosol, activating cGAS-STING and NLRP3 inflammasome, leading to increased SASP and ROS production. Mitophagy reduction, such as PTEN-induced kinase 1 (PINK1)/Parkin, impairs the clearance of damaged mitochondria, while chronic activation of poly ADP ribose polymerase (PARP) in response to DNA damage depletes NAD^+^ and further compromises respiratory reserve capacity [127,128,129,130]. NAD homeostasis is additionally eroded by ecto- and endo-enzymes such as CD38, and by reduced salvage through Nicotinamide adenine dinucleotide (NAMPT), linking damage signaling to sirtuin-dependent chromatin regulation [131]. Beyond mitophagy, selective autophagy of the endoplasmic reticulum, lysosomes, and peroxisomes, together with TFEB and TFE3-driven lysosome biogenesis, shapes organelle quality and influences whether senescence remains stable or transitions toward degeneration [132,133]. A mitochondrial stress-biased program, often termed MiDAS, can produce p21-high and p16-variable arrest with a distinct secretory signature, demonstrating that not all senescence is DDR-centric [120].

## 3. Senescence and Oxidative Stress in Cardiac Disease

Cardiac disease is a leading global cause of death and disability. Both its prevalence and mortality increase with population aging [134]. Age is the most common independent risk factor for cardiac disease and is accompanied by impaired cardiac structure and function [135]. Rarely dividing/post-mitotic cardiomyocytes exhibit a senescence-like phenotype (reduced metabolic flexibility, Ca^2+^ handling, mitochondrial dysfunction, and increased inflammatory signaling) that contributes to age-related functional decline [18,136,137]. In addition, hemodynamic overload, ischemia–reperfusion, and metabolic dysfunction (e.g., insulin resistance and lipotoxicity) promote oxidative stress and accelerate cardiac senescence [18,138]. Although the molecular mechanism underlying oxidative stress-induced cardiac senescence is not fully understood, elevated ROS levels are a consistent feature of cardiac senescence [139,140]. In the cardiac senescent cell, mitochondrial Ca^2^⁺ overload triggers the opening of the mitochondrial permeability transition pore (mPTP), loss of ΔΨm, and ATP depletion, while impaired PINK1/Parkin-mediated mitophagy, lysosomal deacidification, and reduced TFEB signaling delay the clearance of damaged mitochondria [141,142,143]. Consequently, mtDNA deletions and point mutations accumulate with age, while impaired mitochondrial quality control elevates ROS with positive feedback [139,144]. In these sections, we examine the mechanistic contribution of the senescence–oxidative stress axis to the pathogenesis of major cardiac diseases.

### 3.1. Ischemia–Reperfusion Injury

During ischemia, mETC activity is inhibited, and succinate accumulates [145]. During reperfusion, oxidation of succinate triggers reverse electron transport (RET) at complex I and causes increased mitochondrial ROS (mtROS) [146]. Subsequently, cytosolic and mitochondrial Ca^2+^ overload, opening of mPTP, loss of ΔΨm, and ATP depletion occur, thereby increasing cardiac injury [141,147]. Nevertheless, both the identification of mPTP and the proposed distinction that transient opening is physiological and sustained opening is pathological remain controversial [142]. Previous studies have reported that cardiomyocytes, endothelial cells, and fibroblasts at the infarct border zone exhibit increased p16 and p21 expression accompanied by SASP accumulation [148,149,150,151]. Importantly, these cell types exhibit distinct SASP profiles that differentially impact post-ischemic remodeling. Senescent cardiomyocytes are a primary source of CCN1 (cyr61) and pro-fibrotic factors such as TGF-β, which drive replacement fibrosis. In contrast, senescent cardiac fibroblast and endothelial cells predominantly secrete pro-inflammatory cytokines (e.g., IL-6 and IL-1β) and MMPs, which perpetuate inflammation and degrade the extracellular matrix. Consistent with the relative resistance of senescent cells to apoptosis, experimental models indicate that senescent cardiomyocytes and non-myocytes are not efficiently eliminated during I/R, resulting in persistence and accumulation within the peri-infarct region. Genetic and pharmacological senolytic strategies that increase apoptotic susceptibility in senescent cells, such as BCL-2 family inhibition with navitoclax or combined dasatinib and quercetin, reduce senescent cell accumulation, attenuate adverse remodeling, and improve post-infarction cardiac function in aged mice, indicating that senescent cells survive the initial ischemic insult and contribute to chronic injury [152,153]. However, whether these signatures are causal drivers or downstream consequences of injury remains unclear and appears to depend on the experimental model and the assay used [154]. Recent studies indicated that lipid peroxidation-dependent ferroptosis contributes to I/R injury [155,156]. In addition, mtDNA release with the activation of the cGAS/STING pathway can exacerbate inflammation after reperfusion [157,158]. I/R-induced senescent cardiomyocyte has been linked to hypertrophy, inflammation, and fibrosis via SASP mediators, although some studies report that senescence induction in fibroblasts limits fibrosis and promote repair, suggesting dependence on cell type, timing, and experimental conditions [154,159,160]. Clinical trials of broad antioxidant supplementation have not shown consistent benefits, and low-level ROS may mediate preconditioning, indicating the importance of dose and timing [161,162,163,164,165]. More targeted strategies administered at reperfusion appear promising [166,167]. In experimental models, the administration of malonate at reperfusion to inhibit succinate oxidation attenuates injury and adverse remodeling [146,165]. In contrast, acute inhibition of monocarboxylate transporter 1 (MCT1) to prevent succinate efflux has been reported to increase mtROS and exacerbate injury [168]. These findings emphasize the importance of the RET and succinate timing axis. Overall, I/R injury may be mitigated by approaches that attenuate the early increase in ROS driven by RET, prevent the sustained opening of mPTP, restore mitochondrial quality control, and preserve NAD⁺ metabolism. The modulation of ferroptosis and the cGAS/STING pathway should be guided by the prevailing phenotype. Success is likely to depend on biomarker-guided phenotyping using measures such as succinate and oxidized phospholipids, mitochondrial damage-associated molecular patterns derived from mtDNA, lipid peroxidation markers, and cellular senescence markers, together with precise therapeutic timing.

### 3.2. Heart Failure with Preserved Ejection Fraction (HFpEF)

Heart failure with preserved ejection fraction is a heterogeneous syndrome prevalent among older adults with obesity, type 2 diabetes, and hypertension [169,170]. Systemic inflammation and coronary microvascular dysfunction are frequent. Endothelial oxidative stress attenuates NO/cyclic guanosine monophosphate (cGMP)/protein kinase G (PKG) signaling, which reduces titin phosphorylation, increases passive myocardial stiffness, and impairs diastolic relaxation [171,172]. Disruptions to redox and signaling pathways occur within a cellular environment characterized by senescence across multiple cardiac cell types [173,174]. In the coronary microvasculature, endothelial cells exhibit increased p16 and p21, loss of LMNB1, telomere-associated DNA damage foci, and SASP enriched in IL-1β, IL-6, TGF-β, and matrix metalloproteinases (MMPs) [173,175]. Consequently, SASP factors promote microvascular rarefaction, endothelial-to-mesenchymal transition (EndMT), and interstitial remodeling [176,177]. In addition, senescent cardiac fibroblasts promote collagen deposition and cross-linking, whereas cardiomyocytes exhibit mitochondrial dysfunction, impaired mitophagy, reduced NAD⁺ and sirtuin activity, and metabolic inflexibility [178,179,180,181]. Consequently, senescence pathways interact with redox imbalance and reduced NO/cGMP/PKG signaling to enhance diastolic dysfunction [182,183]. Despite the established preliminary model, robust experimental validation for the proposed interaction has yet to be provided. Clinically, senescence signatures are heterogeneous and depend on the analytic method, tissue accessibility, and biomarker specificity [2,184]. Currently, available circulating markers remain nonspecific, while the relative contribution of vascular and extracardiac drivers remains controversial, and the causal relationship between microvascular senescence and clinical HFpEF phenotypes has not been definitively established [185,186,187]. Clinical trials that directly augmented the NO/cGMP/PKG pathway have yielded neutral or mixed results, likely reflecting heterogeneity in pathway predominance by patient phenotype, intervention timing, and comorbid state [188,189,190]. By contrast, interventions that attenuate metabolic, inflammatory, and oxidative stress have shown benefit. Sodium–glucose cotransporter-2 (SGLT-2) inhibitors reduce HF in preserved or mildly reduced ejection fraction, and glucagon-like peptide-1 (GLP-1) receptor agonists improve symptoms, physical function, and weight in obese phenotypes [191,192,193,194]. Senescence-targeted therapies, including senolytics and senostatics, remain experimental, with human efficacy and safety still uncertain. Taken together, HFpEF reflects an interplay among microvascular endothelial senescence with oxidative stress, reduced NO/cGMP/PKG signaling, impaired mitochondrial quality control, and metabolic dysfunction. Given that dominant mechanisms are not uniform across patients, treatment should be phenotype-based. Weight reduction combined with SGLT-2 inhibitors or GLP-1 receptor agonists is appropriate in obese metabolic–inflammatory phenotypes. Approaches that enhance microvascular function and NO production are reasonable in cases characterized by predominant fibrosis. Biomarker panels and imaging of microvascular function will be essential for precise phenotyping and therapy.

### 3.3. Dilated Cardiomyopathy

Dilated cardiomyopathy involves chronic oxidative stress, defective organelle quality control, and the accumulation of senescence markers in cardiomyocytes and cardiac fibroblasts [195,196,197]. Mitochondrial dysfunction, with impaired mitophagy, lysosomal deacidification, and reduced TFEB signaling, disrupts ATP homeostasis and increases ROS [198,199,200]. Proteostasis failure driven by loss-of-function variants in titin and mutations in lamin A/C (LMNA), BCL2-associated athanogene 3 (BAG3), and desmin (DES) produces proteotoxic and ER stress with depleted cellular NAD⁺ [201,202,203,204,205]. Nuclear envelope instability and micronuclei formation lead to cytosolic DNA release and an activated cGAS/STING pathway, sustaining inflammatory signaling and SASP, which promotes adverse remodeling, systolic dysfunction, and an arrhythmogenic substrate [75,206]. Nevertheless, senescence markers are not consistently detected in detailed cardiomyopathy patients; concurrently, antioxidant supplementation has failed to yield consistent benefits in clinical trials [2,207]. NOX isoforms may fulfill conflicting roles across varying physiological contexts, evidenced by reports of both detrimental and adaptive signaling [208,209,210,211]. The penetrance of titin-truncating variants remains unresolved, with competing attributions to haploinsufficiency, poison-peptide mechanism, and additional second hits such as viral myocarditis, pressure overload, and metabolic stress [212,213]. Although senolytic and autophagy-enhancing approaches have shown promise in preclinical studies, efficacy and safety in human dilated cardiomyopathy have not yet been demonstrated [214,215]. Overall, dilated cardiomyopathy reflects an interplay between redox imbalance, failures in organelle quality control, and stress-responsive transcriptional programs. Therapeutic development should emphasize phenotype-guided approaches that target mitochondrial function and mitophagy, stabilize proteostasis, modulate cGAS/STING signaling, and support NAD⁺ metabolism, together with guideline-directed neurohormonal blockade.

### 3.4. Cardiac Hypertrophy and Remodeling

**Figure 2:**
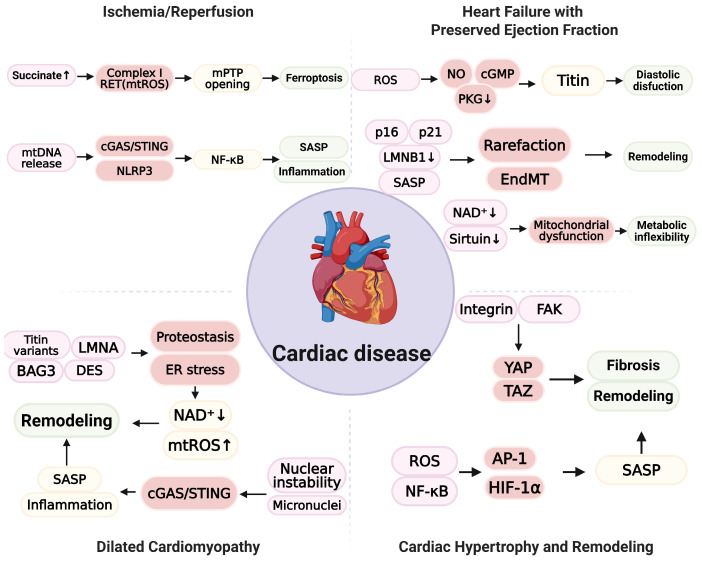
Schematic representation of senescence-oxidative stress coupling across cardiac disease. In ischemia/reperfusion (I/R), succinate accumulation drives complex I reverse electron transport (RET) and increases mtROS, thereby promoting the opening of mitochondrial permeability transition pore (mPTP) and ferroptosis. mtDNA release activates cGAS/STING and NLRP3, initiates NF-κB signaling, and augments SASP and inflammation, contributing to injury and early remodeling. In heart failure with preserved ejection fraction (HFpEF), endothelial oxidative stress depresses NO/cGMP/PKG signaling, reduces titin compliance, and produces diastolic dysfunction. Endothelial p16 and p21, together with loss of LMNB1 and SASP, promote microvascular rarefaction and endothelial-to-mesenchymal transition (EndMT), leading to interstitial remodeling. In cardiomyocytes, depletion of NAD⁺ and reduced sirtuin activity are associated with mitochondrial dysfunction and metabolic inflexibility. In dilated cardiomyopathy, variants in titin, LMNA, BAG3, and DES disrupt proteostasis and induce ER stress with NAD⁺ depletion and increased mtROS. Nuclear instability and micronuclei activate cGAS/STING, amplify SASP and inflammation, and drive adverse remodeling. In cardiac hypertrophy and remodeling, mechanical or metabolic load is signaled via integrin and focal adhesion kinase (FAK) to yes-associated protein/transcriptional coactivator with PDZ-binding motif (YAP/TAZ) and promotes fibrosis and remodeling. ROS activate NF-κB, AP-1, and HIF-1α, increase SASP, and further contribute to remodeling. (Figure created in Biorender. Hyeong Rok Yun. (2025) https://app.biorender.com/illustrations/canvas-beta/690b558c0fc19569ee5e6335).

Cardiac hypertrophy is an initially adaptive response to hemodynamic or metabolic load but often progresses to maladaptive remodeling with fibrosis, microvascular rarefaction, and diastolic or systolic dysfunction [216]. Recent studies indicate that senescence-oxidative stress crosstalk is central to driving progression from adaptive hypertrophy to pathological remodeling [18,217]. Mechanical and metabolic stress increase mtROS and activate redox-sensitive transcriptional programs such as NF-κB, activating protein-1 (AP-1), and hypoxia-inducible factor-1 alpha (HIF-1α), together with mechanotransduction pathways including integrin–focal adhesion kinase (FAK) and yes-associated protein/transcriptional coactivator with PDZ-binding motif (YAP/TAZ) [218,219,220,221]. Taz acts with the TEA domain (TEAD) to convert mechanical load into pro-hypertrophic and pro-fibrotic gene expression [222]. Perturbed Ca^2+^ handling at mitochondria–ER contact sites promotes mitochondrial permeability transition, loss of ΔΨm, and energetic inefficiency [223]. Single-cell and spatial profiling reveal heterogeneous senescent states across endothelial cells, fibroblasts, and cardiomyocytes [224]. Distinct SASP profiles among these cell populations dictate a specific therapeutic target. For example, senescent cardiomyocytes secrete endothelin-1(EDN-1) and GDF15, which act in an autocrine/paracrine manner to promote hypertrophy and fibrosis, whereas senescent fibroblasts exhibit a strongly inflammatory SASP (IL-6 and CCL2) that recruits immune cells. Endothelial p16 and p21 expression with SASP enriched in adhesion molecules (ICAM-1 and VCAM-1) and TGF-β promote EndMT and microvascular rarefaction [175,225]. Fibroblast populations range from proinflammatory, matrix-secreting cells to quiescent, matrifibrocyte-like states, suggesting time- and cell-type-specific contributions of senescence to remodeling [226,227]. Cytosolic mtDNA and micronuclei activate cGAS/STING and inflammasome pathways, thereby linking organelle injury to sterile inflammation and fibrotic progression [228,229]. However, several crucial mechanistic aspects have yet to be fully elucidated. Physiologically, low levels of ROS mediate adaptive signaling, complicating antioxidant strategies [230]. The consequences of YAP/TAZ signaling depend on the cellular state and timing—early after load, transient YAP/TAZ activation is protective—whereas sustained activation drives fibrotic remodeling [222,231,232]. In addition, whether senescence in cardiomyocytes or fibroblasts precedes remodeling or activates downstream consequences remains unknown, and the direction of the effect likely varies with the model, timing, and measurement endpoints [233]. Therapeutic strategies for hypertrophic remodeling should emphasize unloading and stiffness reduction, together with the selective modulation of mechanotransduction pathways [234,235]. The strategy comprises augmentation of cGMP/protein kinase G (PKG) signaling to increase titin compliance, targeting integrin–FAK and YAP/TAZ to suppress prohypertrophic/profibrotic programs, anti-fibrotic strategies that limit fibroblast activation and extracellular-matrix cross-linking, improvement in microvascular function, and isoform-specific load-dependent modulation of NOX activity [236,237,238]. Responses should be evaluated using remodeling-specific endpoints, such as left ventricular mass, extracellular volume fraction, and myocardial strain, to guide biomarker-informed, stage-specific interventions [239]. An overview of cardiac disease is shown in Figure 2.

### 3.5. Congenital Heart Disease

Congenital heart disease (CHD) represents a complex pathophysiological paradigm in which chronic hypoxia and hemodynamic overload accelerate biological aging through distinct molecular mechanisms [240,241]. Unlike HF in adults, the myocardium in CHD is exposed to sustained cyanosis and pressure loading from early development, leading to maladaptive metabolic reprogramming driven by hypoxia-inducible factor-1α (HIF-1α) [242,243]. While initially adaptive, chronic HIF-1α stabilization alters mitochondrial biogenetics and upregulates NOX2 and NOX4, thereby establishing a persistent source of mitochondrial and cytosolic ROS [244,245]. Under these conditions, NRF2 signaling is insufficient to yield an adequate antioxidant response, resulting in chronic redox dysfunction and exacerbated oxidative injury [246,247]. Cumulative oxidative stress directly compromises genomic stability. ROS-induced DNA double-stranded breaks activate the ATM/ATR-p53-p21 axis, initiating cell-cycle arrest, while concurrent oxidative damage to telomeres suppresses telomerase reverse transcriptase (TERT) activity, thereby accelerating telomere shortening in cardiomyocytes and vascular endothelial cells [248,249]. Crucially, the transition from damage accumulation to a stable senescent state is consolidated by p16 upregulation, which correlates with ventricular dysfunction severity in patients with CHD. Senescent cells adopt SASP characterized by NF-κB-dependent secretion of IL-6, TGF-β, and MMPs, which drives EndMT and myocardial fibrosis [250]. Furthermore, perioperative I/R injury and mechanical stress from cardiopulmonary bypass invoke marked increases in mtROS and the release of mtDNA, which may trigger the cGAS/STING pathway and link metabolic stress to sterile inflammation [157,214,251]. Thus, CHD constitutes a trajectory of “accelerated cardiovascular aging” driven by the convergence of hypoxic signaling, NRF2 dysfunction, and p53/p16-mediated senescence. 

### 3.6. Clinical Translation and Ongoing Trials Targeting the Senescence-Oxidative Stress Axis

Notwithstanding the robust preclinical evidence elucidating the therapeutic potential of the senescence-oxidative stress axis, clinical translation remains in its early stages [252]. Contemporary clinical investigations are primarily stratified into two distinct approaches: the repurposing of established pharmacotherapies with pleiotropic senomorphic properties and the evaluation of novel senolytic or mitochondria-targeted agents [17,139]. The most substantial clinical advancement has been realized with sodium-glucose cotransporter-2 (SGLT-2) inhibitors such as Empagliflozin and Dapagliflozin. Although initially developed as antidiabetic agents, landmark cardiovascular outcome trials have substantiated efficacy in reducing HF hospitalization and mortality across all ejection fraction ranges [192,253]. Mechanistically, these agents have been demonstrated to attenuate oxidative stress, ameliorate mitochondrial energetics, and suppress cellular senescence pathways in cardiomyocytes and endothelial cells, thereby effectively functioning as putative “senomorphics” in clinical practice [254,255]. Concurrently, Metformin is under active investigation for the potential to alleviate age-related cardiovascular decline via AMPK activation and ROS reduction, and the prospective TAME (Targeting Aging with Metformin) trial is anticipated to provide definitive insights [252,256]. Pharmacological strategies targeting the selective elimination of senescent cells, termed senolytics, are currently subject to evaluation in pilot clinical trials. The combination of Dasatinib and Quercetin is being assessed for safety and efficacy in pathologies exhibiting significant mechanistic overlap with adverse cardiac remodeling, including diabetic kidney disease and idiopathic pulmonary fibrosis [257]. Furthermore, the flavonoid Fisetin is under investigation in the AFFIRM-LITE trial to delineate its impact on frailty and systemic inflammation in the geriatric population [258,259]. While these agents exhibit therapeutic promise, clinical application necessitates precise dosing protocols to optimize the therapeutic window while minimizing off-target cytotoxicity. In parallel, interventions designed to directly modulate mitochondrial oxidative stress and antioxidant defense mechanisms have yielded heterogeneous outcomes. Elamipretide (SS-31), a peptide engineered to stabilize cardiolipin and reduce mtROS, has been evaluated in HF cohorts [139,141]. Although broad efficacy endpoints have shown variability, post hoc analyses suggest potential benefits in patient subgroups defined by specific mitochondrial phenotypes. Additionally, NRF2 activators such as bardoxolone methyl have demonstrated the capacity to augment antioxidant response elements in renal pathologies, although the cardiovascular safety profile necessitates vigilant monitoring due to potential fluid retention [260]. Collectively, these translational efforts underscore a paradigm shift toward precision medicine and necessitate the stratification of therapeutic interventions based on individual oxidative or senescent phenotypes.

## 4. Conclusions

The cellular senescence–oxidative stress axis represents an extensive pathophysiological mechanism in cardiac disease. Understanding integrated signaling pathways enables the development of target-, timing-, and phenotype-guided approaches for prevention and therapy. Recent studies focus on senolytic and senostatic strategies. In the future, advances in multi-omics (metabolomics, proteomics, and genomics) and precision biomarker development will permit precise identification and stratification of patients characterized by a predominant senescence-oxidative stress axis, enabling mechanism-informed, individualized therapy. Outstanding priorities include elucidating cell-type and temporal dependencies, establishing causal mechanisms, and undertaking adequately powered systemic human studies to define safety and efficacy.

## Data Availability

No new data were created or analyzed in this study. Data sharing is not applicable to this article.

## Abbreviations

AKT	Protein kinase B
AP-1	Activator protein-1
APC/C	Anaphase-promoting complex/cyclosome
ATM	Ataxia–telangiectasia mutated
ATP	Adenosine triphosphate
ATR	ATM- and Rad3-related
BACH1	BTB and CNC homology 1
BAG3	BCL2-associated athanogene 3
BCL-2	B-cell lymphoma-2
BET	Bromodomain and extraterminal (family)
BRD4	Bromodomain-containing protein 4
C/EBPβ	CCAAT/enhancer-binding protein-β
CDH1	APC/C co-activator CDC20 homolog 1
CDK2/4/6	Cyclin-dependent kinase 2/4/6
CDKN1A (p21)	Cyclin-dependent kinase inhibitor 1A
CDKN2A (p16)	Cyclin-dependent kinase inhibitor 2A
cGAS	Cyclic GMP–AMP synthase
cGMP	Cyclic guanosine monophosphate
CUL3	Cullin 3
DNA-SCARS	DNA segments with chromatin alterations reinforcing senescence
DP	Dimerization partner (of E2F)
DREAM	DP, RB-like, E2F, and MuvB complex
DRP1	Dynamin-related protein-1
DUOX1/2	Dual oxidase 1/2
E2F	E2 promoter-binding factor
ECM	Extracellular matrix
eIF2α	Eukaryotic initiation factor-2 alpha
EndMT	Endothelial-to-mesenchymal transition
EP300 (p300)	E1A-binding protein p300
ER	Endoplasmic reticulum
FAK	Focal adhesion kinase
GLP-1	Glucagon-like peptide-1
GPx4	Glutathione peroxidase-4
GPxs	Glutathione peroxidases
GR	Glutathione reductase
GSH	Reduced glutathione
H_2_O_2_	Hydrogen peroxide
H3K9me3	Histone H3 lysine-9 trimethylation
HF	Heart failure
HFpEF	Heart failure with preserved ejection fraction
HIF-1α	Hypoxia-inducible factor-1 alpha
HMGB2	High-mobility group box 2
HMOX1	Heme oxygenase-1
HO•	Hydroxyl radical
HOCl	Hypochlorous acid
HP1	Heterochromatin protein-1
I/R	Ischemia–reperfusion
IFN	Interferon (type I unless otherwise specified)
IL-1β	Interleukin-1 beta
IL-6	Interleukin-6
IL-8	Interleukin-8
JAK1/2	Janus kinase-1/2
KEAP1	Kelch-like ECH-associated protein-1
LMNA	Lamin A/C
LMNB1	Lamin B1
macroH2A	Macro-H2A histone variant
MAPK	Mitogen-activated protein kinase
MCP-1	Monocyte chemoattractant protein-1
MCT1	Monocarboxylate transporter-1
MDA-5	Melanoma differentiation-associated gene-5
mETC	Mitochondrial electron transport chain
MFN1/2	Mitofusin-1/2
MiDAS	Mitochondrial dysfunction-associated senescence
MK2	MAPK-activated protein kinase-2
MMPs	Matrix metalloproteinases
mPTP	Mitochondrial permeability transition pore
mRNA	Messenger RNA
mtDNA	Mitochondrial DNA
mTOR	Mechanistic target of rapamycin
mTORC1	Mechanistic target of rapamycin complex-1
mtROS	Mitochondrial reactive oxygen species
NAD⁺	Oxidized nicotinamide adenine dinucleotide
NADPH	Nicotinamide adenine dinucleotide phosphate
NAMPT	Nicotinamide phosphoribosyltransferase
NF-κB	Nuclear factor-κB
NO	Nitric oxide
NOSs	Nitric oxide synthases
NOX	NADPH oxidase
NOX2	NADPH oxidase-2
NRF2	Nuclear factor erythroid 2-related factor-2
O_2_•^−^	Superoxide
ONOO^−^	Peroxynitrite
OPA1	Optic atrophy-1
PARP	Poly(ADP-ribose) polymerase
PI3K	Phosphatidylinositol-3-kinase
PINK1	PTEN-induced kinase-1
PKG	Protein kinase-G
Prxs	Peroxiredoxins
PUFA	Polyunsaturated fatty acid
RB	Retinoblastoma protein
RET	Reverse electron transport
RIG-I	Retinoic acid-inducible gene-I
RNS	Reactive nitrogen species
ROH	Lipid alcohol
ROO•	Lipid peroxyl radical
ROOH	Lipid hydroperoxide
ROS	Reactive oxygen species
SA-β-gal	Senescence-associated β-galactosidase
SAHFs	Senescence-associated heterochromatin foci
SAMD	Senescence-associated mitochondrial dysfunction
SASP	Senescence-associated secretory phenotype
SGLT-2	Sodium–glucose cotransporter-2
SIRT(s)	Sirtuin(s)
SOD1/2	Superoxide dismutase-1/2
STING	Stimulator of interferon genes
TAZ	Transcriptional co-activator with PDZ-binding motif
TEAD	TEA domain transcription factor
TFE3	Transcription factor E3
TFEB	Transcription factor EB
TGF-β	Transforming growth factor-β
TLR-3	Toll-like receptor-3
Trx	Thioredoxin
YAP	Yes-associated protein
ΔΨm	Mitochondrial membrane potential

## References

[1] Ajoolabady A., Pratico D., Bahijri S., Tuomilehto J., Uversky V.N., Ren J. (2025). Hallmarks of cellular senescence: Biology, mechanisms, regulations. Exp. Mol. Med..

[2] Ogrodnik M., Carlos Acosta J., Adams P.D., d’Adda di Fagagna F., Baker D.J., Bishop C.L., Chandra T., Collado M., Gil J., Gorgoulis V. (2024). Guidelines for minimal information on cellular senescence experimentation in vivo. Cell.

[3] Hayflick L., Moorhead P.S. (1961). The serial cultivation of human diploid cell strains. Exp. Cell Res..

[4] Wang B., Han J., Elisseeff J.H., Demaria M. (2024). The senescence-associated secretory phenotype and its physiological and pathological implications. Nat. Rev. Mol. Cell Biol..

[5] Acosta J.C., Banito A., Wuestefeld T., Georgilis A., Janich P., Morton J.P., Athineos D., Kang T.W., Lasitschka F., Andrulis M. (2013). A complex secretory program orchestrated by the inflammasome controls paracrine senescence. Nat. Cell Biol..

[6] Liao Z., Yeo H.L., Wong S.W., Zhao Y. (2021). Cellular Senescence: Mechanisms and Therapeutic Potential. Biomedicines.

[7] Coppe J.P., Desprez P.Y., Krtolica A., Campisi J. (2010). The senescence-associated secretory phenotype: The dark side of tumor suppression. Annu. Rev. Pathol. Mech. Dis..

[8] Lagares D., Santos A., Grasberger P.E., Liu F., Probst C.K., Rahimi R.A., Sakai N., Kuehl T., Ryan J., Bhola P. (2017). Targeted apoptosis of myofibroblasts with the BH3 mimetic ABT-263 reverses established fibrosis. Sci. Transl. Med..

[9] Munoz-Espin D., Canamero M., Maraver A., Gomez-Lopez G., Contreras J., Murillo-Cuesta S., Rodriguez-Baeza A., Varela-Nieto I., Ruberte J., Collado M. (2013). Programmed cell senescence during mammalian embryonic development. Cell.

[10] Storer M., Mas A., Robert-Moreno A., Pecoraro M., Ortells M.C., Di Giacomo V., Yosef R., Pilpel N., Krizhanovsky V., Sharpe J. (2013). Senescence is a developmental mechanism that contributes to embryonic growth and patterning. Cell.

[11] Demaria M., Ohtani N., Youssef S.A., Rodier F., Toussaint W., Mitchell J.R., Laberge R.M., Vijg J., Van Steeg H., Dolle M.E. (2014). An essential role for senescent cells in optimal wound healing through secretion of PDGF-AA. Dev. Cell.

[12] Fraga C.G., Shigenaga M.K., Park J.W., Degan P., Ames B.N. (1990). Oxidative damage to DNA during aging: 8-hydroxy-2’-deoxyguanosine in rat organ DNA and urine. Proc. Natl. Acad. Sci. USA.

[13] Li B., Ming H., Qin S., Nice E.C., Dong J., Du Z., Huang C. (2025). Redox regulation: Mechanisms, biology and therapeutic targets in diseases. Signal Transduct. Target. Ther..

[14] Atayik M.C., Cakatay U. (2023). Redox signaling and modulation in ageing. Biogerontology.

[15] Goyal P., Maurer M.S., Roh J. (2024). Aging in Heart Failure: Embracing Biology Over Chronology: JACC Family Series. JACC Heart Fail..

[16] Forman D.E., de Lemos J.A., Shaw L.J., Reuben D.B., Lyubarova R., Peterson E.D., Spertus J.A., Zieman S., Salive M.E., Rich M.W. (2020). Cardiovascular Biomarkers and Imaging in Older Adults: JACC Council Perspectives. J. Am. Coll. Cardiol..

[17] Abdellatif M., Rainer P.P., Sedej S., Kroemer G. (2023). Hallmarks of cardiovascular ageing. Nat. Rev. Cardiol..

[18] Grootaert M.O.J. (2024). Cell senescence in cardiometabolic diseases. NPJ Aging.

[19] Brandl A., Meyer M., Bechmann V., Nerlich M., Angele P. (2011). Oxidative stress induces senescence in human mesenchymal stem cells. Exp. Cell Res..

[20] d’Adda di Fagagna F., Reaper P.M., Clay-Farrace L., Fiegler H., Carr P., Von Zglinicki T., Saretzki G., Carter N.P., Jackson S.P. (2003). A DNA damage checkpoint response in telomere-initiated senescence. Nature.

[21] Serrano M., Lin A.W., McCurrach M.E., Beach D., Lowe S.W. (1997). Oncogenic ras provokes premature cell senescence associated with accumulation of p53 and p16INK4a. Cell.

[22] Chondrogianni N., Stratford F.L., Trougakos I.P., Friguet B., Rivett A.J., Gonos E.S. (2003). Central role of the proteasome in senescence and survival of human fibroblasts: Induction of a senescence-like phenotype upon its inhibition and resistance to stress upon its activation. J. Biol. Chem..

[23] Gallage S., Gil J. (2016). Mitochondrial Dysfunction Meets Senescence. Trends Biochem. Sci..

[24] Canman C.E., Lim D.S., Cimprich K.A., Taya Y., Tamai K., Sakaguchi K., Appella E., Kastan M.B., Siliciano J.D. (1998). Activation of the ATM kinase by ionizing radiation and phosphorylation of p53. Science.

[25] Zhao H., Piwnica-Worms H. (2001). ATR-mediated checkpoint pathways regulate phosphorylation and activation of human Chk1. Mol. Cell. Biol..

[26] el-Deiry W.S., Tokino T., Velculescu V.E., Levy D.B., Parsons R., Trent J.M., Lin D., Mercer W.E., Kinzler K.W., Vogelstein B. (1993). WAF1, a potential mediator of p53 tumor suppression. Cell.

[27] Freund A., Patil C.K., Campisi J. (2011). p38MAPK is a novel DNA damage response-independent regulator of the senescence-associated secretory phenotype. EMBO J..

[28] Bracken A.P., Kleine-Kohlbrecher D., Dietrich N., Pasini D., Gargiulo G., Beekman C., Theilgaard-Monch K., Minucci S., Porse B.T., Marine J.C. (2007). The Polycomb group proteins bind throughout the INK4A-ARF locus and are disassociated in senescent cells. Genes Dev..

[29] Xiong Y., Hannon G.J., Zhang H., Casso D., Kobayashi R., Beach D. (1993). p21 is a universal inhibitor of cyclin kinases. Nature.

[30] Serrano M., Hannon G.J., Beach D. (1993). A new regulatory motif in cell-cycle control causing specific inhibition of cyclin D/CDK4. Nature.

[31] Buchkovich K., Duffy L.A., Harlow E. (1989). The retinoblastoma protein is phosphorylated during specific phases of the cell cycle. Cell.

[32] Quelle D.E., Zindy F., Ashmun R.A., Sherr C.J. (1995). Alternative reading frames of the INK4a tumor suppressor gene encode two unrelated proteins capable of inducing cell cycle arrest. Cell.

[33] Charrier-Savournin F.B., Chateau M.T., Gire V., Sedivy J., Piette J., Dulic V. (2004). p21-Mediated nuclear retention of cyclin B1-Cdk1 in response to genotoxic stress. Mol. Biol. Cell.

[34] Lossaint G., Horvat A., Gire V., Bacevic K., Mrouj K., Charrier-Savournin F., Georget V., Fisher D., Dulic V. (2022). Reciprocal regulation of p21 and Chk1 controls the cyclin D1-RB pathway to mediate senescence onset after G2 arrest. J. Cell Sci..

[35] Uxa S., Bernhart S.H., Mages C.F.S., Fischer M., Kohler R., Hoffmann S., Stadler P.F., Engeland K., Muller G.A. (2019). DREAM and RB cooperate to induce gene repression and cell-cycle arrest in response to p53 activation. Nucleic Acids Res..

[36] Fischer M., Quaas M., Steiner L., Engeland K. (2016). The p53-p21-DREAM-CDE/CHR pathway regulates G2/M cell cycle genes. Nucleic Acids Res..

[37] Engeland K. (2022). Cell cycle regulation: p53-p21-RB signaling. Cell Death Differ..

[38] Wei W., Ayad N.G., Wan Y., Zhang G.J., Kirschner M.W., Kaelin W.G. (2004). Degradation of the SCF component Skp2 in cell-cycle phase G1 by the anaphase-promoting complex. Nature.

[39] Listovsky T., Sale J.E. (2013). Sequestration of CDH1 by MAD2L2 prevents premature APC/C activation prior to anaphase onset. J. Cell Biol..

[40] Mouery B.L., Baker E.M., Mei L., Wolff S.C., Mills C.A., Fleifel D., Mulugeta N., Herring L.E., Cook J.G. (2024). APC/C prevents a noncanonical order of cyclin/CDK activity to maintain CDK4/6 inhibitor-induced arrest. Proc. Natl. Acad. Sci. USA.

[41] Koliopoulos M.G., Alfieri C. (2022). Cell cycle regulation by complex nanomachines. FEBS J..

[42] Purvis J.E., Karhohs K.W., Mock C., Batchelor E., Loewer A., Lahav G. (2012). p53 dynamics control cell fate. Science.

[43] Yosef R., Pilpel N., Tokarsky-Amiel R., Biran A., Ovadya Y., Cohen S., Vadai E., Dassa L., Shahar E., Condiotti R. (2016). Directed elimination of senescent cells by inhibition of BCL-W and BCL-XL. Nat. Commun..

[44] Sohn D., Essmann F., Schulze-Osthoff K., Janicke R. (2006). U. p21 blocks irradiation-induced apoptosis downstream of mitochondria by inhibition of cyclin-dependent kinase-mediated caspase-9 activation. Cancer Res..

[45] Wang R., Yu Z., Sunchu B., Shoaf J., Dang I., Zhao S., Caples K., Bradley L., Beaver L.M., Ho E. (2017). Rapamycin inhibits the secretory phenotype of senescent cells by a Nrf2-independent mechanism. Aging Cell.

[46] Xu M., Tchkonia T., Ding H., Ogrodnik M., Lubbers E.R., Pirtskhalava T., White T.A., Johnson K.O., Stout M.B., Mezera V. (2015). JAK inhibition alleviates the cellular senescence-associated secretory phenotype and frailty in old age. Proc. Natl. Acad. Sci. USA.

[47] Tasdemir N., Banito A., Roe J.S., Alonso-Curbelo D., Camiolo M., Tschaharganeh D.F., Huang C.H., Aksoy O., Bolden J.E., Chen C.C. (2016). BRD4 Connects Enhancer Remodeling to Senescence Immune Surveillance. Cancer Discov..

[48] Yang H., Wang H., Ren J., Chen Q., Chen Z.J. (2017). cGAS is essential for cellular senescence. Proc. Natl. Acad. Sci. USA.

[49] Hoare M., Ito Y., Kang T.W., Weekes M.P., Matheson N.J., Patten D.A., Shetty S., Parry A.J., Menon S., Salama R. (2016). NOTCH1 mediates a switch between two distinct secretomes during senescence. Nat. Cell Biol..

[50] Kang C., Xu Q., Martin T.D., Li M.Z., Demaria M., Aron L., Lu T., Yankner B.A., Campisi J., Elledge S.J. (2015). The DNA damage response induces inflammation and senescence by inhibiting autophagy of GATA4. Science.

[51] Takai H., Smogorzewska A., de Lange T. (2003). DNA damage foci at dysfunctional telomeres. Curr. Biol..

[52] Hewitt G., Jurk D., Marques F.D., Correia-Melo C., Hardy T., Gackowska A., Anderson R., Taschuk M., Mann J., Passos J.F. (2012). Telomeres are favoured targets of a persistent DNA damage response in ageing and stress-induced senescence. Nat. Commun..

[53] Rodier F., Munoz D.P., Teachenor R., Chu V., Le O., Bhaumik D., Coppe J.P., Campeau E., Beausejour C.M., Kim S.H. (2011). DNA-SCARS: Distinct nuclear structures that sustain damage-induced senescence growth arrest and inflammatory cytokine secretion. J. Cell Sci..

[54] Bester A.C., Roniger M., Oren Y.S., Im M.M., Sarni D., Chaoat M., Bensimon A., Zamir G., Shewach D.S., Kerem B. (2011). Nucleotide deficiency promotes genomic instability in early stages of cancer development. Cell.

[55] Jung S.H., Hwang H.J., Kang D., Park H.A., Lee H.C., Jeong D., Lee K., Park H.J., Ko Y.G., Lee J.S. (2019). mTOR kinase leads to PTEN-loss-induced cellular senescence by phosphorylating p53. Oncogene.

[56] Alimonti A., Nardella C., Chen Z., Clohessy J.G., Carracedo A., Trotman L.C., Cheng K., Varmeh S., Kozma S.C., Thomas G. (2010). A novel type of cellular senescence that can be enhanced in mouse models and human tumor xenografts to suppress prostate tumorigenesis. J. Clin. Investig..

[57] Wiley C.D., Campisi J. (2021). The metabolic roots of senescence: Mechanisms and opportunities for intervention. Nat. Metab..

[58] Mercurio L., Bailey J., Glick A.B., Dellambra E., Scarponi C., Pallotta S., Albanesi C., Madonna S. (2024). RAS-activated PI3K/AKT signaling sustains cellular senescence via P53/P21 axis in experimental models of psoriasis. J. Dermatol. Sci..

[59] Di Micco R., Fumagalli M., Cicalese A., Piccinin S., Gasparini P., Luise C., Schurra C., Garre M., Nuciforo P.G., Bensimon A. (2006). Oncogene-induced senescence is a DNA damage response triggered by DNA hyper-replication. Nature.

[60] Kotsantis P., Silva L.M., Irmscher S., Jones R.M., Folkes L., Gromak N., Petermann E. (2016). Increased global transcription activity as a mechanism of replication stress in cancer. Nat. Commun..

[61] Donne R., Saroul-Ainama M., Cordier P., Hammoutene A., Kabore C., Stadler M., Nemazanyy I., Galy-Fauroux I., Herrag M., Riedl T. (2022). Replication stress triggered by nucleotide pool imbalance drives DNA damage and cGAS-STING pathway activation in NAFLD. Dev. Cell.

[62] Kuilman T., Michaloglou C., Vredeveld L.C., Douma S., van Doorn R., Desmet C.J., Aarden L.A., Mooi W.J., Peeper D.S. (2008). Oncogene-induced senescence relayed by an interleukin-dependent inflammatory network. Cell.

[63] Coppe J.P., Patil C.K., Rodier F., Sun Y., Munoz D.P., Goldstein J., Nelson P.S., Desprez P.Y., Campisi J. (2008). Senescence-associated secretory phenotypes reveal cell-nonautonomous functions of oncogenic RAS and the p53 tumor suppressor. PLoS Biol..

[64] Acosta J.C., O’Loghlen A., Banito A., Guijarro M.V., Augert A., Raguz S., Fumagalli M., Da Costa M., Brown C., Popov N. (2008). Chemokine signaling via the CXCR2 receptor reinforces senescence. Cell.

[65] Ozcan S., Alessio N., Acar M.B., Mert E., Omerli F., Peluso G., Galderisi U. (2016). Unbiased analysis of senescence associated secretory phenotype (SASP) to identify common components following different genotoxic stresses. Aging.

[66] Laberge R.M., Sun Y., Orjalo A.V., Patil C.K., Freund A., Zhou L., Curran S.C., Davalos A.R., Wilson-Edell K.A., Liu S. (2015). MTOR regulates the pro-tumorigenic senescence-associated secretory phenotype by promoting IL1A translation. Nat. Cell Biol..

[67] Teo Y.V., Rattanavirotkul N., Olova N., Salzano A., Quintanilla A., Tarrats N., Kiourtis C., Muller M., Green A.R., Adams P.D. (2019). Notch Signaling Mediates Secondary Senescence. Cell Rep..

[68] Tiedje C., Ronkina N., Tehrani M., Dhamija S., Laass K., Holtmann H., Kotlyarov A., Gaestel M. (2012). The p38/MK2-driven exchange between tristetraprolin and HuR regulates AU-rich element-dependent translation. PLoS Genet..

[69] Narita M., Nunez S., Heard E., Narita M., Lin A.W., Hearn S.A., Spector D.L., Hannon G.J., Lowe S.W. (2003). Rb-mediated heterochromatin formation and silencing of E2F target genes during cellular senescence. Cell.

[70] Zhang R., Poustovoitov M.V., Ye X., Santos H.A., Chen W., Daganzo S.M., Erzberger J.P., Serebriiskii I.G., Canutescu A.A., Dunbrack R.L. (2005). Formation of MacroH2A-containing senescence-associated heterochromatin foci and senescence driven by ASF1a and HIRA. Dev. Cell.

[71] Contrepois K., Thuret J.Y., Courbeyrette R., Fenaille F., Mann C. (2012). Deacetylation of H4-K16Ac and heterochromatin assembly in senescence. Epigenet. Chromatin.

[72] Ivanov A., Pawlikowski J., Manoharan I., van Tuyn J., Nelson D.M., Rai T.S., Shah P.P., Hewitt G., Korolchuk V.I., Passos J.F. (2013). Lysosome-mediated processing of chromatin in senescence. J. Cell Biol..

[73] Sadaie M., Salama R., Carroll T., Tomimatsu K., Chandra T., Young A.R., Narita M., Perez-Mancera P.A., Bennett D.C., Chong H. (2013). Redistribution of the Lamin B1 genomic binding profile affects rearrangement of heterochromatic domains and SAHF formation during senescence. Genes Dev..

[74] Aird K.M., Iwasaki O., Kossenkov A.V., Tanizawa H., Fatkhutdinov N., Bitler B.G., Le L., Alicea G., Yang T.L., Johnson F.B. (2016). HMGB2 orchestrates the chromatin landscape of senescence-associated secretory phenotype gene loci. J. Cell Biol..

[75] Mackenzie K.J., Carroll P., Martin C.A., Murina O., Fluteau A., Simpson D.J., Olova N., Sutcliffe H., Rainger J.K., Leitch A. (2017). cGAS surveillance of micronuclei links genome instability to innate immunity. Nature.

[76] De Cecco M., Ito T., Petrashen A.P., Elias A.E., Skvir N.J., Criscione S.W., Caligiana A., Brocculi G., Adney E.M., Boeke J.D. (2019). L1 drives IFN in senescent cells and promotes age-associated inflammation. Nature.

[77] Mullani N., Porozhan Y., Mangelinck A., Rachez C., Costallat M., Batsche E., Goodhardt M., Cenci G., Mann C., Muchardt C. (2021). Reduced RNA turnover as a driver of cellular senescence. Life Sci. Alliance.

[78] Kim D., Chen R., Sheu M., Kim N., Kim S., Islam N., Wier E.M., Wang G., Li A., Park A. (2019). Noncoding dsRNA induces retinoic acid synthesis to stimulate hair follicle regeneration via TLR3. Nat. Commun..

[79] Lu A.T., Quach A., Wilson J.G., Reiner A.P., Aviv A., Raj K., Hou L., Baccarelli A.A., Li Y., Stewart J.D. (2019). DNA methylation GrimAge strongly predicts lifespan and healthspan. Aging.

[80] Garcia-Prat L., Martinez-Vicente M., Perdiguero E., Ortet L., Rodriguez-Ubreva J., Rebollo E., Ruiz-Bonilla V., Gutarra S., Ballestar E., Serrano A.L. (2016). Autophagy maintains stemness by preventing senescence. Nature.

[81] Dimri G.P., Lee X., Basile G., Acosta M., Scott G., Roskelley C., Medrano E.E., Linskens M., Rubelj I., Pereira-Smith O. (1995). A biomarker that identifies senescent human cells in culture and in aging skin in vivo. Proc. Natl. Acad. Sci. USA.

[82] Kakimoto Y., Okada C., Kawabe N., Sasaki A., Tsukamoto H., Nagao R., Osawa M. (2019). Myocardial lipofuscin accumulation in ageing and sudden cardiac death. Sci. Rep..

[83] Young A.R., Narita M., Ferreira M., Kirschner K., Sadaie M., Darot J.F., Tavare S., Arakawa S., Shimizu S., Watt F.M. (2009). Autophagy mediates the mitotic senescence transition. Genes Dev..

[84] Hara T., Nakamura K., Matsui M., Yamamoto A., Nakahara Y., Suzuki-Migishima R., Yokoyama M., Mishima K., Saito I., Okano H. (2006). Suppression of basal autophagy in neural cells causes neurodegenerative disease in mice. Nature.

[85] Harding H.P., Zhang Y., Bertolotti A., Zeng H., Ron D. (2000). Perk is essential for translational regulation and cell survival during the unfolded protein response. Mol. Cell.

[86] Hamanaka R.B., Bennett B.S., Cullinan S.B., Diehl J.A. (2005). PERK and GCN2 contribute to eIF2alpha phosphorylation and cell cycle arrest after activation of the unfolded protein response pathway. Mol. Biol. Cell.

[87] Kang E., Kang C., Lee Y.S., Lee S.V. (2024). Brief guide to senescence assays using cultured mammalian cells. Mol. Cells.

[88] Jeon O.H., Wilson D.R., Clement C.C., Rathod S., Cherry C., Powell B., Lee Z., Khalil A.M., Green J.J., Campisi J. (2019). Senescence cell-associated extracellular vesicles serve as osteoarthritis disease and therapeutic markers. JCI Insight.

[89] Seara F.A.C., Maciel L., Kasai-Brunswick T.H., Nascimento J.H.M., Campos-de-Carvalho A.C. (2023). Extracellular Vesicles and Cardiac Aging. Adv. Exp. Med. Biol..

[90] Hu Y., Xue X., Han T., Li Y., Zhang T., Lu T., Zhang P. (2025). An effective system for senescence modulating drug development using quantitative high-content analysis and high-throughput screening. Commun. Biol..

[91] di Candia A.M., de Avila D.X., Moreira G.R., Villacorta H., Maisel A.S. (2021). Growth differentiation factor-15, a novel systemic biomarker of oxidative stress, inflammation, and cellular aging: Potential role in cardiovascular diseases. Am. Heart J. Plus Cardiol. Res. Pract..

[92] Murphy M.P., Bayir H., Belousov V., Chang C.J., Davies K.J.A., Davies M.J., Dick T.P., Finkel T., Forman H.J., Janssen-Heininger Y. (2022). Guidelines for measuring reactive oxygen species and oxidative damage in cells and in vivo. Nat. Metab..

[93] Chandimali N., Bak S.G., Park E.H., Lim H.J., Won Y.S., Kim E.K., Park S.I., Lee S.J. (2025). Free radicals and their impact on health and antioxidant defenses: A review. Cell Death Discov..

[94] Hunt M., Torres M., Bachar-Wikstrom E., Wikstrom J.D. (2024). Cellular and molecular roles of reactive oxygen species in wound healing. Commun. Biol..

[95] Okado-Matsumoto A., Fridovich I. (2001). Subcellular distribution of superoxide dismutases (SOD) in rat liver: Cu,Zn-SOD in mitochondria. J. Biol. Chem..

[96] Zheng Y., Sun J., Luo Z., Li Y., Huang Y. (2024). Emerging mechanisms of lipid peroxidation in regulated cell death and its physiological implications. Cell Death Dis..

[97] Duche G., Sanderson J.M. (2024). The Chemical Reactivity of Membrane Lipids. Chem. Rev..

[98] Kim J.W., Lee J.Y., Oh M., Lee E.W. (2023). An integrated view of lipid metabolism in ferroptosis revisited via lipidomic analysis. Exp. Mol. Med..

[99] Noreng S., Ota N., Sun Y., Ho H., Johnson M., Arthur C.P., Schneider K., Lehoux I., Davies C.W., Mortara K. (2022). Structure of the core human NADPH oxidase NOX2. Nat. Commun..

[100] Zheng M., Liu Y., Zhang G., Yang Z., Xu W., Chen Q. (2023). The Applications and Mechanisms of Superoxide Dismutase in Medicine, Food, and Cosmetics. Antioxidants.

[101] Chen Y.S., Tian H.X., Rong D.C., Wang L., Chen S., Zeng J., Xu H., Mei J., Wang L.Y., Liou Y.L. (2025). ROS homeostasis in cell fate, pathophysiology, and therapeutic interventions. Mol. Biomed..

[102] Kumar N., He J., Rusling J.F. (2023). Electrochemical transformations catalyzed by cytochrome P450s and peroxidases. Chem. Soc. Rev..

[103] Lai W., Zhang J., Sun J., Min T., Bai Y., He J., Cao H., Che Q., Guo J., Su Z. (2024). Oxidative stress in alcoholic liver disease, focusing on proteins, nucleic acids, and lipids: A review. Int. J. Biol. Macromol..

[104] Jomova K., Alomar S.Y., Valko R., Fresser L., Nepovimova E., Kuca K., Valko M. (2025). Interplay of oxidative stress and antioxidant mechanisms in cancer development and progression. Arch. Toxicol..

[105] Kohda A., Kamakura S., Hayase J., Sumimoto H. (2024). The NADPH oxidases DUOX1 and DUOX2 are sorted to the apical plasma membrane in epithelial cells via their respective maturation factors DUOXA1 and DUOXA2. Genes Cells.

[106] Jomova K., Raptova R., Alomar S.Y., Alwasel S.H., Nepovimova E., Kuca K., Valko M. (2023). Reactive oxygen species, toxicity, oxidative stress, and antioxidants: Chronic diseases and aging. Arch. Toxicol..

[107] Wang W., Liang L., Dai Z., Zuo P., Yu S., Lu Y., Ding D., Chen H., Shan H., Jin Y. (2024). A conserved N-terminal motif of CUL3 contributes to assembly and E3 ligase activity of CRL3(KLHL22). Nat. Commun..

[108] Horie Y., Suzuki T., Inoue J., Iso T., Wells G., Moore T.W., Mizushima T., Dinkova-Kostova A.T., Kasai T., Kamei T. (2021). Molecular basis for the disruption of Keap1-Nrf2 interaction via Hinge & Latch mechanism. Commun. Biol..

[109] Reichard J.F., Motz G.T., Puga A. (2007). Heme oxygenase-1 induction by NRF2 requires inactivation of the transcriptional repressor BACH1. Nucleic Acids Res..

[110] Chen J.H., Hales C.N., Ozanne S.E. (2007). DNA damage, cellular senescence and organismal ageing: Causal or correlative?. Nucleic Acids Res..

[111] Guan J., Li T., Ma F., Wang N., Zhang H., Li J., Li J., Xu C., Liu Q. (2025). DNA damage-dependent mechanisms of ionizing radiation-induced cellular senescence. PeerJ.

[112] Chien Y., Scuoppo C., Wang X., Fang X., Balgley B., Bolden J.E., Premsrirut P., Luo W., Chicas A., Lee C.S. (2011). Control of the senescence-associated secretory phenotype by NF-kappaB promotes senescence and enhances chemosensitivity. Genes Dev..

[113] Flanagan K.C., Alspach E., Pazolli E., Parajuli S., Ren Q., Arthur L.L., Tapia R., Stewart S.A. (2018). c-Myb and C/EBPbeta regulate OPN and other senescence-associated secretory phenotype factors. Oncotarget.

[114] Baird L., Taguchi K., Zhang A., Takahashi Y., Suzuki T., Kensler T.W., Yamamoto M. (2023). A NRF2-induced secretory phenotype activates immune surveillance to remove irreparably damaged cells. Redox Biol..

[115] Hedblom A., Hejazi S.M., Canesin G., Choudhury R., Hanafy K.A., Csizmadia E., Persson J.L., Wegiel B. (2019). Heme detoxification by heme oxygenase-1 reinstates proliferative and immune balances upon genotoxic tissue injury. Cell Death Dis..

[116] Cuollo L., Antonangeli F., Santoni A., Soriani A. (2020). The Senescence-Associated Secretory Phenotype (SASP) in the Challenging Future of Cancer Therapy and Age-Related Diseases. Biology.

[117] Cogliati S., Frezza C., Soriano M.E., Varanita T., Quintana-Cabrera R., Corrado M., Cipolat S., Costa V., Casarin A., Gomes L.C. (2013). Mitochondrial cristae shape determines respiratory chain supercomplexes assembly and respiratory efficiency. Cell.

[118] Pedrera L., Prieto Clemente L., Dahlhaus A., Lotfipour Nasudivar S., Tishina S., Olmo Gonzalez D., Stroh J., Yapici F.I., Singh R.P., Grotehans N. (2025). Ferroptosis triggers mitochondrial fragmentation via Drp1 activation. Cell Death Dis..

[119] Camacho-Encina M., Booth L.K., Redgrave R.E., Folaranmi O., Spyridopoulos I., Richardson G.D. (2024). Cellular Senescence, Mitochondrial Dysfunction, and Their Link to Cardiovascular Disease. Cells.

[120] Wiley C.D., Velarde M.C., Lecot P., Liu S., Sarnoski E.A., Freund A., Shirakawa K., Lim H.W., Davis S.S., Ramanathan A. (2016). Mitochondrial Dysfunction Induces Senescence with a Distinct Secretory Phenotype. Cell Metab..

[121] Zhang X., Gao Y., Zhang S., Wang Y., Pei X., Chen Y., Zhang J., Zhang Y., Du Y., Hao S. (2025). Mitochondrial dysfunction in the regulation of aging and aging-related diseases. Cell Commun. Signal..

[122] Han X., Li W., He X., Lu X., Zhang Y., Li Y., Bi G., Ma X., Huang X., Bai R. (2023). Blockade of TGF-beta signalling alleviates human adipose stem cell senescence induced by native ECM in obesity visceral white adipose tissue. Stem Cell Res. Ther..

[123] Miwa S., Kashyap S., Chini E., von Zglinicki T. (2022). Mitochondrial dysfunction in cell senescence and aging. J. Clin. Investig..

[124] Ong S.B., Samangouei P., Kalkhoran S.B., Hausenloy D.J. (2015). The mitochondrial permeability transition pore and its role in myocardial ischemia reperfusion injury. J. Mol. Cell. Cardiol..

[125] Protasoni M., Serrano M. (2023). Targeting Mitochondria to Control Ageing and Senescence. Pharmaceutics.

[126] Kwong J.Q., Molkentin J.D. (2015). Physiological and pathological roles of the mitochondrial permeability transition pore in the heart. Cell Metab..

[127] Shimada K., Crother T.R., Karlin J., Dagvadorj J., Chiba N., Chen S., Ramanujan V.K., Wolf A.J., Vergnes L., Ojcius D.M. (2012). Oxidized mitochondrial DNA activates the NLRP3 inflammasome during apoptosis. Immunity.

[128] Zhong Z., Liang S., Sanchez-Lopez E., He F., Shalapour S., Lin X.J., Wong J., Ding S., Seki E., Schnabl B. (2018). New mitochondrial DNA synthesis enables NLRP3 inflammasome activation. Nature.

[129] Matsuda N., Sato S., Shiba K., Okatsu K., Saisho K., Gautier C.A., Sou Y.S., Saiki S., Kawajiri S., Sato F. (2010). PINK1 stabilized by mitochondrial depolarization recruits Parkin to damaged mitochondria and activates latent Parkin for mitophagy. J. Cell Biol..

[130] Bai P., Canto C., Oudart H., Brunyanszki A., Cen Y., Thomas C., Yamamoto H., Huber A., Kiss B., Houtkooper R.H. (2011). PARP-1 inhibition increases mitochondrial metabolism through SIRT1 activation. Cell Metab..

[131] Camacho-Pereira J., Tarrago M.G., Chini C.C.S., Nin V., Escande C., Warner G.M., Puranik A.S., Schoon R.A., Reid J.M., Galina A. (2016). CD38 Dictates Age-Related NAD Decline and Mitochondrial Dysfunction through an SIRT3-Dependent Mechanism. Cell Metab..

[132] Settembre C., Di Malta C., Polito V.A., Garcia Arencibia M., Vetrini F., Erdin S., Erdin S.U., Huynh T., Medina D., Colella P. (2011). TFEB links autophagy to lysosomal biogenesis. Science.

[133] Martina J.A., Diab H.I., Brady O.A., Puertollano R. (2016). TFEB and TFE3 are novel components of the integrated stress response. EMBO J..

[134] Chong B., Jayabaskaran J., Jauhari S.M., Chan S.P., Goh R., Kueh M.T.W., Li H., Chin Y.H., Kong G., Anand V.V. (2025). Global burden of cardiovascular diseases: Projections from 2025 to 2050. Eur. J. Prev. Cardiol..

[135] Vakka A., Warren J.S., Drosatos K. (2023). Cardiovascular aging: From cellular and molecular changes to therapeutic interventions. J. Cardiovasc. Aging.

[136] Zhang H., Muhetarijiang M., Chen R.J., Hu X., Han J., Zheng L., Chen T. (2024). Mitochondrial Dysfunction: A Roadmap for Understanding and Tackling Cardiovascular Aging. Aging Dis..

[137] Ali M.A., Gioscia-Ryan R., Yang D., Sutton N.R., Tyrrell D.J. (2024). Cardiovascular aging: Spotlight on mitochondria. Am. J. Physiol. Heart Circ. Physiol..

[138] Zhai P., Sadoshima J. (2024). Cardiomyocyte senescence and the potential therapeutic role of senolytics in the heart. J. Cardiovasc. Aging.

[139] Zong Y., Li H., Liao P., Chen L., Pan Y., Zheng Y., Zhang C., Liu D., Zheng M., Gao J. (2024). Mitochondrial dysfunction: Mechanisms and advances in therapy. Signal Transduct. Target. Ther..

[140] Zhang S.Y., Yang Y.H., Wen R., Yang N., Feng S.S., Zhang T.N. (2025). Cellular and molecular mechanisms underlying cardiovascular aging. Cell. Mol. Biol. Lett..

[141] Bertero E., Popoiu T.A., Maack C. (2024). Mitochondrial calcium in cardiac ischemia/reperfusion injury and cardioprotection. Basic Res. Cardiol..

[142] Bernardi P., Gerle C., Halestrap A.P., Jonas E.A., Karch J., Mnatsakanyan N., Pavlov E., Sheu S.S., Soukas A.A. (2023). Identity, structure, and function of the mitochondrial permeability transition pore: Controversies, consensus, recent advances, and future directions. Cell Death Differ..

[143] Yoshii A., McMillen T.S., Wang Y., Zhou B., Chen H., Banerjee D., Herrero M., Wang P., Muraoka N., Wang W. (2024). Blunted Cardiac Mitophagy in Response to Metabolic Stress Contributes to HFpEF. Circ. Res..

[144] Green A.P., Klimm F., Marshall A.S., Leetmaa R., Aryaman J., Gomez-Duran A., Chinnery P.F., Jones N.S. (2025). Cryptic mitochondrial DNA mutations coincide with mid-late life and are pathophysiologically informative in single cells across tissues and species. Nat. Commun..

[145] Chouchani E.T., Pell V.R., Gaude E., Aksentijevic D., Sundier S.Y., Robb E.L., Logan A., Nadtochiy S.M., Ord E.N.J., Smith A.C. (2014). Ischaemic accumulation of succinate controls reperfusion injury through mitochondrial ROS. Nature.

[146] Abe J., Vujic A., Prag H.A., Murphy M.P., Krieg T. (2024). Malonate given at reperfusion prevents post-myocardial infarction heart failure by decreasing ischemia/reperfusion injury. Basic Res. Cardiol..

[147] Ashok D., Papanicolaou K., Sidor A., Wang M., Solhjoo S., Liu T., O’Rourke B. (2023). Mitochondrial membrane potential instability on reperfusion after ischemia does not depend on mitochondrial Ca^2+^ uptake. J. Biol. Chem..

[148] Cui S., Xue L., Yang F., Dai S., Han Z., Liu K., Liu B., Yuan Q., Cui Z., Zhang Y. (2018). Postinfarction Hearts Are Protected by Premature Senescent Cardiomyocytes Via GATA 4-Dependent CCN 1 Secretion. J. Am. Heart Assoc..

[149] Baggett B.C., Murphy K.R., Sengun E., Mi E., Cao Y., Turan N.N., Lu Y., Schofield L., Kim T.Y., Kabakov A.Y. (2023). Myofibroblast senescence promotes arrhythmogenic remodeling in the aged infarcted rabbit heart. eLife.

[150] Wang P., Konja D., Singh S., Zhang B., Wang Y. (2024). Endothelial Senescence: From Macro- to Micro-Vasculature and Its Implications on Cardiovascular Health. Int. J. Mol. Sci..

[151] Jin M., Li C., Wu Z., Tang Z., Xie J., Wei G., Yang Z., Huang S., Chen Y., Li X. (2025). Inhibiting the Histone Demethylase Kdm4a Restrains Cardiac Fibrosis After Myocardial Infarction by Promoting Autophagy in Premature Senescent Fibroblasts. Adv. Sci..

[152] Suda M., Paul K.H., Minamino T., Miller J.D., Lerman A., Ellison-Hughes G.M., Tchkonia T., Kirkland J.L. (2023). Senescent Cells: A Therapeutic Target in Cardiovascular Diseases. Cells.

[153] Walaszczyk A., Dookun E., Redgrave R., Tual-Chalot S., Victorelli S., Spyridopoulos I., Owens A., Arthur H.M., Passos J.F., Richardson G.D. (2019). Pharmacological clearance of senescent cells improves survival and recovery in aged mice following acute myocardial infarction. Aging Cell.

[154] Redgrave R.E., Dookun E., Booth L.K., Camacho Encina M., Folaranmi O., Tual-Chalot S., Gill J.H., Owens W.A., Spyridopoulos I., Passos J.F. (2023). Senescent cardiomyocytes contribute to cardiac dysfunction following myocardial infarction. npj Aging.

[155] Wang X., Chen T., Chen S., Zhang J., Cai L., Liu C., Zhang Y., Wu X., Li N., Ma Z. (2025). STING aggravates ferroptosis-dependent myocardial ischemia-reperfusion injury by targeting GPX4 for autophagic degradation. Signal Transduct. Target. Ther..

[156] Cai W., Liu L., Shi X., Liu Y., Wang J., Fang X., Chen Z., Ai D., Zhu Y., Zhang X. (2023). Alox15/15-HpETE Aggravates Myocardial Ischemia-Reperfusion Injury by Promoting Cardiomyocyte Ferroptosis. Circulation.

[157] Xiong Y., Leng Y., Tian H., Deng X., Li W., Li W., Xia Z. (2023). Decreased MFN2 activates the cGAS-STING pathway in diabetic myocardial ischaemia-reperfusion by triggering the release of mitochondrial DNA. Cell Commun. Signal..

[158] Cao D.J., Schiattarella G.G., Villalobos E., Jiang N., May H.I., Li T., Chen Z.J., Gillette T.G., Hill J.A. (2018). Cytosolic DNA Sensing Promotes Macrophage Transformation and Governs Myocardial Ischemic Injury. Circulation.

[159] Feng T., Meng J., Kou S., Jiang Z., Huang X., Lu Z., Zhao H., Lau L.F., Zhou B., Zhang H. (2019). CCN1-Induced Cellular Senescence Promotes Heart Regeneration. Circulation.

[160] Meyer K., Hodwin B., Ramanujam D., Engelhardt S., Sarikas A. (2016). Essential Role for Premature Senescence of Myofibroblasts in Myocardial Fibrosis. J. Am. Coll. Cardiol..

[161] Ye Y., Li J., Yuan Z. (2013). Effect of antioxidant vitamin supplementation on cardiovascular outcomes: A meta-analysis of randomized controlled trials. PLoS ONE.

[162] Dhalla N.S., Ostadal P., Tappia P.S. (2025). Involvement of Oxidative Stress and Antioxidants in Modification of Cardiac Dysfunction Due to Ischemia-Reperfusion Injury. Antioxidants.

[163] Kiely M., Brennan L., Dunlop T., Tate G., Woodside J.V., Moretti D., Working Group 1 of the Federation of European Nutrition Societies (FENS) Presidential Activity (2025). Prevention of chronic disease using vitamins-a case study of the vitamin D and cardiovascular disease hypothesis using evidence from randomised controlled and prospective cohort studies. Eur. J. Nutr..

[164] Pagliaro P., Alloatti G., Penna C. (2025). Cardioprotection Reloaded: Reflections on 40 Years of Research. Antioxidants.

[165] Valls-Lacalle L., Consegal M., Ganse F.G., Yanez-Bisbe L., Pastor J., Ruiz-Meana M., Inserte J., Benito B., Ferreira-Gonzalez I., Rodriguez-Sinovas A. (2024). Long-Term Protective Effects of Succinate Dehydrogenase Inhibition during Reperfusion with Malonate on Post-Infarction Left Ventricular Scar and Remodeling in Mice. Int. J. Mol. Sci..

[166] Prag H.A., Aksentijevic D., Dannhorn A., Giles A.V., Mulvey J.F., Sauchanka O., Du L., Bates G., Reinhold J., Kula-Alwar D. (2022). Ischemia-Selective Cardioprotection by Malonate for Ischemia/Reperfusion Injury. Circ. Res..

[167] Chahardehi A.M., Arefnezhad R., Rafei S., Arzhangzadeh A., Nasiri R., Rezaei-Tazangi F., Tavakoli M.R. (2025). The Effect of Malonate as a Succinate Dehydrogenase Inhibitor on Myocardial Ischemia/Reperfusion Injury. Cell Biol. Int..

[168] Milliken A.S., Nadtochiy S.M., Brookes P.S. (2022). Inhibiting Succinate Release Worsens Cardiac Reperfusion Injury by Enhancing Mitochondrial Reactive Oxygen Species Generation. J. Am. Heart Assoc..

[169] Borlaug B.A., Sharma K., Shah S.J., Ho J.E. (2023). Heart Failure with Preserved Ejection Fraction: JACC Scientific Statement. J. Am. Coll. Cardiol..

[170] Paolisso P., Gallinoro E., Belmonte M., Bertolone D.T., Bermpeis K., De Colle C., Shumkova M., Leone A., Caglioni S., Esposito G. (2024). Coronary Microvascular Dysfunction in Patients with Heart Failure: Characterization of Patterns in HFrEF Versus HFpEF. Circ. Heart Fail..

[171] Rosas P.C., Neves L.A.A., Patel N., Tran D., Pereira C.H., Bonilla K.R., Zheng J., Sun J., Alvarado F.J., Banach K. (2024). Early pathological mechanisms in a mouse model of heart failure with preserved ejection fraction. Am. J. Physiol. Heart Circ. Physiol..

[172] Hamdani N., Bishu K.G., von Frieling-Salewsky M., Redfield M.M., Linke W.A. (2013). Deranged myofilament phosphorylation and function in experimental heart failure with preserved ejection fraction. Cardiovasc. Res..

[173] Gevaert A.B., Shakeri H., Leloup A.J., Van Hove C.E., De Meyer G.R.Y., Vrints C.J., Lemmens K., Van Craenenbroeck E.M. (2017). Endothelial Senescence Contributes to Heart Failure with Preserved Ejection Fraction in an Aging Mouse Model. Circ. Heart Fail..

[174] Roh J., Hill J.A., Singh A., Valero-Muñoz M., Sam F. (2022). Heart Failure with Preserved Ejection Fraction: Heterogeneous Syndrome, Diverse Preclinical Models. Circ. Res..

[175] Bloom S.I., Islam M.T., Lesniewski L.A., Donato A.J. (2023). Mechanisms and consequences of endothelial cell senescence. Nat. Rev. Cardiol..

[176] Ye B., Bradshaw A.D., Abrahante J.E., Dragon J.A., Haussler T.N., Bell S.P., Hirashima F., LeWinter M., Zile M.R., Meyer M. (2023). Left Ventricular Gene Expression in Heart Failure with Preserved Ejection Fraction-Profibrotic and Proinflammatory Pathways and Genes. Circ. Heart Fail..

[177] Romano E., Rosa I., Fioretto B.S., Manetti M. (2024). The contribution of endothelial cells to tissue fibrosis. Curr. Opin. Rheumatol..

[178] Li H., Zhu X., Cao X., Lu Y., Zhou J., Zhang X. (2023). Single-cell analysis reveals lysyl oxidase (Lox)^+^ fibroblast subset involved in cardiac fibrosis of diabetic mice. J. Adv. Res..

[179] Yang J., Savvatis K., Kang J.S., Fan P., Zhong H., Schwartz K., Barry V., Mikels-Vigdal A., Karpinski S., Kornyeyev D. (2016). Targeting LOXL2 for cardiac interstitial fibrosis and heart failure treatment. Nat. Commun..

[180] Sun N., Barta H., Chaudhuri S., Chen K., Jin J., Luo H., Yang M., Krigman J., Zhang R., Sanghvi S. (2025). Mitophagy mitigates mitochondrial fatty acid beta-oxidation deficient cardiomyopathy. Nat. Commun..

[181] Ding Y.N., Wang H.Y., Chen X.F., Tang X., Chen H.Z. (2025). Roles of Sirtuins in Cardiovascular Diseases: Mechanisms and Therapeutics. Circ. Res..

[182] Herzog M.J., Muller P., Lechner K., Stiebler M., Arndt P., Kunz M., Ahrens D., Schmeisser A., Schreiber S., Braun-Dullaeus R.C. (2025). Arterial stiffness and vascular aging: Mechanisms, prevention, and therapy. Signal Transduct. Target. Ther..

[183] Daou D., Gillette T.G., Hill J.A. (2023). Inflammatory Mechanisms in Heart Failure with Preserved Ejection Fraction. Physiology.

[184] Gurkar A.U., Gerencser A.A., Mora A.L., Nelson A.C., Zhang A.R., Lagnado A.B., Enninful A., Benz C., Furman D., Beaulieu D. (2023). Spatial mapping of cellular senescence: Emerging challenges and opportunities. Nat. Aging.

[185] La Vecchia G., Fumarulo I., Caffe A., Chiatto M., Montone R.A., Aspromonte N. (2024). Microvascular Dysfunction across the Spectrum of Heart Failure Pathology: Pathophysiology, Clinical Features and Therapeutic Implications. Int. J. Mol. Sci..

[186] Sen P., Wang L., d’Ambrosio L., Bierschenk S., Hamers J., Ornek I., Sittig T., Zhang H., Zhang J., Merkus D. (2025). Coronary microvascular disease in heart failure with preserved ejection fraction. Physiol. Rep..

[187] Weerts J., Schroen B.L.M., Barandiaran Aizpurua A., Berendschot T., Brandts L., Webers C.A.B., Simons S.O., Meex S.J.R., Henry R., van der Kallen C.J.H. (2025). Microvascular dysfunction across organs in heart failure with preserved ejection fraction: The PROSE-HFpEF case-control study. Cardiovasc. Diabetol..

[188] Redfield M.M., Chen H.H., Borlaug B.A., Semigran M.J., Lee K.L., Lewis G., LeWinter M.M., Rouleau J.L., Bull D.A., Mann D.L. (2013). Effect of phosphodiesterase-5 inhibition on exercise capacity and clinical status in heart failure with preserved ejection fraction: A randomized clinical trial. JAMA.

[189] Redfield M.M., Anstrom K.J., Levine J.A., Koepp G.A., Borlaug B.A., Chen H.H., LeWinter M.M., Joseph S.M., Shah S.J., Semigran M.J. (2015). Isosorbide Mononitrate in Heart Failure with Preserved Ejection Fraction. N. Engl. J. Med..

[190] deFilippi C.R., Shah P., Shah S.J., Alemayehu W., Lam C.S.P., Butler J., Roessig L., O’Connor C.M., Westerhout C.M., Armstrong P.W. (2024). Proteomics Identify Clinical Phenotypes and Predict Functional Outcomes in Heart Failure with Preserved Ejection Fraction: Insights From VITALITY-HFpEF. Circ. Heart Fail..

[191] Anker S.D., Butler J., Filippatos G., Ferreira J.P., Bocchi E., Bohm M., Brunner-La Rocca H.P., Choi D.J., Chopra V., Chuquiure-Valenzuela E. (2021). Empagliflozin in Heart Failure with a Preserved Ejection Fraction. N. Engl. J. Med..

[192] Solomon S.D., McMurray J.J.V., Claggett B., de Boer R.A., DeMets D., Hernandez A.F., Inzucchi S.E., Kosiborod M.N., Lam C.S.P., Martinez F. (2022). Dapagliflozin in Heart Failure with Mildly Reduced or Preserved Ejection Fraction. N. Engl. J. Med..

[193] Kosiborod M.N., Abildstrom S.Z., Borlaug B.A., Butler J., Rasmussen S., Davies M., Hovingh G.K., Kitzman D.W., Lindegaard M.L., Moller D.V. (2023). Semaglutide in Patients with Heart Failure with Preserved Ejection Fraction and Obesity. N. Engl. J. Med..

[194] Kosiborod M.N., Petrie M.C., Borlaug B.A., Butler J., Davies M.J., Hovingh G.K., Kitzman D.W., Moller D.V., Treppendahl M.B., Verma S. (2024). Semaglutide in Patients with Obesity-Related Heart Failure and Type 2 Diabetes. N. Engl. J. Med..

[195] Rouhi L., Auguste G., Zhou Q., Lombardi R., Olcum M., Pourebrahim K., Cheedipudi S.M., Asghar S., Hong K., Robertson M.J. (2022). Deletion of the Lmna gene in fibroblasts causes senescence-associated dilated cardiomyopathy by activating the double-stranded DNA damage response and induction of senescence-associated secretory phenotype. J. Cardiovasc. Aging.

[196] Li B., Xiong W., Zuo W., Shi Y., Wang T., Chang L., Wu Y., Ma H., Bian Q., Chang A.C.Y. (2024). Proximal telomeric decompaction due to telomere shortening drives FOXC1-dependent myocardial senescence. Nucleic Acids Res..

[197] Wang X., Zhang X., Cao K., Zeng M., Fu X., Zheng A., Zhang F., Gao F., Zou X., Li H. (2022). Cardiac disruption of SDHAF4-mediated mitochondrial complex II assembly promotes dilated cardiomyopathy. Nat. Commun..

[198] Li D.L., Wang Z.V., Ding G., Tan W., Luo X., Criollo A., Xie M., Jiang N., May H., Kyrychenko V. (2016). Doxorubicin Blocks Cardiomyocyte Autophagic Flux by Inhibiting Lysosome Acidification. Circulation.

[199] Song R., Lei H., Feng L., Cheng W., Li Y., Yao L.L., Liu J. (2021). TFEB insufficiency promotes cardiac hypertrophy by blocking autophagic degradation of GATA4. J. Biol. Chem..

[200] Yan X., Yang L., Fu X., Luo X., Wang C., Xie Q.P., OuYang F. (2024). Transcription factor EB, a promising therapeutic target in cardiovascular disease. PeerJ.

[201] DePaolo J., Bornstein M., Judy R., Abramowitz S., Verma S.S., Levin M.G., Arany Z., Damrauer S.M. (2024). Titin-Truncating variants Predispose to Dilated Cardiomyopathy in Diverse Populations. medRxiv.

[202] Martin T.G., Pak H., Gerhard G.S., Merali S., Merali C., Lemster B., Dubey P., McTiernan C.F., Bristow M.R., Feldman A.M. (2023). Dysregulated Autophagy and Sarcomere Dysfunction in Patients with Heart Failure with Co-Occurrence of P63A and P380S BAG3 Variants. J. Am. Heart Assoc..

[203] Martin T.G., Myers V.D., Dubey P., Dubey S., Perez E., Moravec C.S., Willis M.S., Feldman A.M., Kirk J.A. (2021). Cardiomyocyte contractile impairment in heart failure results from reduced BAG3-mediated sarcomeric protein turnover. Nat. Commun..

[204] Perry C.E., Halawani S.M., Mukherjee S., Ngaba L.V., Lieu M., Lee W.D., Davis J.G., Adzika G.K., Bebenek A.N., Bazianos D.D. (2024). NAD+ precursors prolong survival and improve cardiac phenotypes in a mouse model of Friedreich’s Ataxia. JCI Insight.

[205] Kedia N., Arhzaouy K., Pittman S.K., Sun Y., Batchelor M., Weihl C.C., Bieschke J. (2019). Desmin forms toxic, seeding-competent amyloid aggregates that persist in muscle fibers. Proc. Natl. Acad. Sci. USA.

[206] Wang W., Gao Y., Lee H.K., Yu A.C., Kipp M., Kaddatz H., Zhan J. (2025). The cGAS/STING Pathway: Friend or Foe in Regulating Cardiomyopathy. Cells.

[207] Salman O., Zamani P., Zhao L., Dib M.J., Gan S., Azzo J.D., Pourmussa B., Richards A.M., Javaheri A., Mann D.L. (2024). Prognostic Significance and Biologic Associations of Senescence-Associated Secretory Phenotype Biomarkers in Heart Failure. J. Am. Heart Assoc..

[208] Ma Y., Zhao H.P., Yang L.G., Li L., Wang A.L., Zhang X.J., Wang K., Yang B., Zhu Z.F., Zhang P.J. (2024). NADPH oxidase 2 mediates cardiac sympathetic denervation and myocyte autophagy, resulting in cardiac atrophy and dysfunction in doxorubicin-induced cardiomyopathy. Sci. Rep..

[209] Zeng H., Zou P., Chen Y., Zhang P., Shao L. (2024). NOX4 aggravates doxorubicin-induced cardiomyocyte pyroptosis by increasing reactive oxygen species content and activating the NLRP3 inflammasome. Cardiovasc. Diagn. Ther..

[210] Vendrov A.E., Xiao H., Lozhkin A., Hayami T., Hu G., Brody M.J., Sadoshima J., Zhang Y.Y., Runge M.S., Madamanchi N.R. (2023). Cardiomyocyte NOX4 regulates resident macrophage-mediated inflammation and diastolic dysfunction in stress cardiomyopathy. Redox Biol..

[211] Labbe P., Thorin E., Thorin-Trescases N. (2025). The Dual Role of NOX4 in Cardiovascular Diseases: Driver of Oxidative Stress and Mediator of Adaptive Remodeling. Antioxidants.

[212] Fomin A., Gartner A., Cyganek L., Tiburcy M., Tuleta I., Wellers L., Folsche L., Hobbach A.J., von Frieling-Salewsky M., Unger A. (2021). Truncated titin proteins and titin haploinsufficiency are targets for functional recovery in human cardiomyopathy due to TTN mutations. Sci. Transl. Med..

[213] Lota A.S., Hazebroek M.R., Theotokis P., Wassall R., Salmi S., Halliday B.P., Tayal U., Verdonschot J., Meena D., Owen R. (2022). Genetic Architecture of Acute Myocarditis and the Overlap with Inherited Cardiomyopathy. Circulation.

[214] Mou J., Chen Y., Zhu X., Xu B., Wang M., Xie J., Lin T., Gu Q., Wu Q., Che Z. (2025). Emerging role of the cGAS-STING pathway in cardiovascular diseases: Biologic function, mechanisms and targeted therapy. Mol. Med..

[215] Li A., Gao M., Liu B., Qin Y., Chen L., Liu H., Wu H., Gong G. (2022). Mitochondrial autophagy: Molecular mechanisms and implications for cardiovascular disease. Cell Death Dis..

[216] Peikert A., Fontana M., Solomon S.D., Thum T. (2025). Left ventricular hypertrophy and myocardial fibrosis in heart failure with preserved ejection fraction: Mechanisms and treatment. Eur. Heart J..

[217] Luan Y., Zhu X., Jiao Y., Liu H., Huang Z., Pei J., Xu Y., Yang Y., Ren K. (2024). Cardiac cell senescence: Molecular mechanisms, key proteins and therapeutic targets. Cell Death Discov..

[218] Xu X., Pang Y., Fan X. (2025). Mitochondria in oxidative stress, inflammation and aging: From mechanisms to therapeutic advances. Signal Transduct. Target. Ther..

[219] Di X., Gao X., Peng L., Ai J., Jin X., Qi S., Li H., Wang K., Luo D. (2023). Cellular mechanotransduction in health and diseases: From molecular mechanism to therapeutic targets. Signal Transduct. Target. Ther..

[220] Elias-Llumbet A., Sharmin R., Berg-Sorensen K., Schirhagl R., Mzyk A. (2024). The Interplay between Mechanoregulation and ROS in Heart Physiology, Disease, and Regeneration. Adv. Heal. Mater..

[221] Cao R., Tian H., Tian Y., Fu X. (2024). A Hierarchical Mechanotransduction System: From Macro to Micro. Adv. Sci..

[222] Song S., Zhang X., Huang Z., Zhao Y., Lu S., Zeng L., Cai F., Wang T., Pei Z., Weng X. (2024). TEA domain transcription factor 1(TEAD1) induces cardiac fibroblasts cells remodeling through BRD4/Wnt4 pathway. Signal Transduct. Target. Ther..

[223] Chen C., Dai G., Fan M., Wang X., Niu K., Gao W. (2025). Mitochondria-associated endoplasmic reticulum membranes and myocardial ischemia: From molecular mechanisms to therapeutic strategies. J. Transl. Med..

[224] Sanborn M.A., Wang X., Gao S., Dai Y., Rehman J. (2025). Unveiling the cell-type-specific landscape of cellular senescence through single-cell transcriptomics using SenePy. Nat. Commun..

[225] Goligorsky M.S. (2024). Permissive role of vascular endothelium in fibrosis: Focus on the kidney. Am. J. Physiol. Physiol..

[226] Patrick R., Janbandhu V., Tallapragada V., Tan S.S.M., McKinna E.E., Contreras O., Ghazanfar S., Humphreys D.T., Murray N.J., Tran Y.T.H. (2024). Integration mapping of cardiac fibroblast single-cell transcriptomes elucidates cellular principles of fibrosis in diverse pathologies. Sci. Adv..

[227] Torimoto K., Elliott K., Nakayama Y., Yanagisawa H., Eguchi S. (2024). Cardiac and perivascular myofibroblasts, matrifibrocytes, and immune fibrocytes in hypertension; commonalities and differences with other cardiovascular diseases. Cardiovasc. Res..

[228] An C., Li Z., Chen Y., Huang S., Yang F., Hu Y., Xu T., Zhang C., Ge S. (2024). The cGAS-STING pathway in cardiovascular diseases: From basic research to clinical perspectives. Cell Biosci..

[229] Liu J., Zhou J., Luan Y., Li X., Meng X., Liao W., Tang J., Wang Z. (2024). cGAS-STING, inflammasomes and pyroptosis: An overview of crosstalk mechanism of activation and regulation. Cell Commun. Signal..

[230] Heusch G., Andreadou I., Bell R., Bertero E., Botker H.E., Davidson S.M., Downey J., Eaton P., Ferdinandy P., Gersh B.J. (2023). Health position paper and redox perspectives on reactive oxygen species as signals and targets of cardioprotection. Redox Biol..

[231] Mia M.M., Cibi D.M., Ghani S., Singh A., Tee N., Sivakumar V., Bogireddi H., Cook S.A., Mao J., Singh M.K. (2022). Loss of Yap/Taz in cardiac fibroblasts attenuates adverse remodelling and improves cardiac function. Cardiovasc. Res..

[232] Ninh V.K., Barlow M., Aydin S., Brand C.S., Yu J., Smith J., Francisco J., Daneman R., King K.R., Miyamoto S. (2025). Cardiomyocyte YAP represses myocardial inflammation and fibrosis and restrains MEF2-regulated gene expression. Am. J. Physiol. Circ. Physiol..

[233] Kumar M., Yan P., Kuchel G.A., Xu M. (2024). Cellular Senescence as a Targetable Risk Factor for Cardiovascular Diseases: Therapeutic Implications: JACC Family Series. JACC Basic Transl. Sci..

[234] Ghazal R., Wang M., Liu D., Tschumperlin D.J., Pereira N.L. (2025). Cardiac Fibrosis in the Multi-Omics Era: Implications for Heart Failure. Circ. Res..

[235] Chalise U., Hale T.M. (2024). Fibroblasts under pressure: Cardiac fibroblast responses to hypertension and antihypertensive therapies. Am. J. Physiol. Heart Circ. Physiol..

[236] Cai Z., Wu C., Xu Y., Cai J., Zhao M., Zu L. (2023). The NO-cGMP-PKG Axis in HFpEF: From Pathological Mechanisms to Potential Therapies. Aging Dis..

[237] Perryman L., Findlay A., Baskar J., Charlton B., Foot J., Hamilton R., Hamprecht D., Joshi A., Stolp J., Turner C. (2025). The small molecule LOXL2 inhibitor SNT-5382 reduces cardiac fibrosis and achieves strong clinical target engagement. Sci. Rep..

[238] Zhao Y., Huang L., Li C., Tang D., Luo Y., Xiang C., Zhou X., Fang J., Wei X., Xia L. (2023). Improvement in coronary microvascular dysfunction evaluated by cardiac magnetic resonance in patients with hypertrophic obstructive cardiomyopathy after transapical beating-heart septal myectomy. Front. Cardiovasc. Med..

[239] Coughlan F., Flynn S., Haenel A., Crilly S., Leipsic J.A., Dodd J.D. (2024). Impactful Cardiac CT and MRI Articles from 2023. Radiol. Cardiothorac. Imaging.

[240] Anjewierden S., O’Sullivan D., Mangold K.E., Attia I.Z., Lopez-Jimenez F., Friedman P.A., Egbe A.C., Connolly H.M., Miranda W.R., Asirvatham S.J. (2025). Artificial Intelligence-Derived Electrocardiographic Age Predicts Mortality in Adults with Congenital Heart Disease. JACC Adv..

[241] Tournoy T.K., Moons P., Daelman B., De Backer J. (2023). Biological Age in Congenital Heart Disease-Exploring the Ticking Clock. J. Cardiovasc. Dev. Dis..

[242] Li Y., Liu X.T., Zhang P.L., Li Y.C., Sun M.R., Wang Y.T., Wang S.P., Yang H., Liu B.L., Wang M. (2022). Hydroxysafflor Yellow A Blocks HIF-1alpha Induction of NOX2 and Protects ZO-1 Protein in Cerebral Microvascular Endothelium. Antioxidants.

[243] Bakleh M.Z., Al Haj Zen A. (2025). The Distinct Role of HIF-1alpha and HIF-2alpha in Hypoxia and Angiogenesis. Cells.

[244] Liu Y., Luo Q., Su Z., Xing J., Wu J., Xiang L., Huang Y., Pan H., Wu X., Zhang X. (2021). Suppression of Myocardial Hypoxia-Inducible Factor-1alpha Compromises Metabolic Adaptation and Impairs Cardiac Function in Patients with Cyanotic Congenital Heart Disease During Puberty. Circulation.

[245] Wienecke L.M., Cohen S., Bauersachs J., Mebazaa A., Chousterman B.G. (2022). Immunity and inflammation: The neglected key players in congenital heart disease?. Heart Fail. Rev..

[246] Gounder S.S., Kannan S., Devadoss D., Miller C.J., Whitehead K.J., Odelberg S.J., Firpo M.A., Paine R., Hoidal J.R., Abel E.D. (2012). Impaired transcriptional activity of Nrf2 in age-related myocardial oxidative stress is reversible by moderate exercise training. PLoS ONE.

[247] Xiong Z., Liao Y., Zhang Z., Wan Z., Liang S., Guo J. (2025). Molecular Insights into Oxidative-Stress-Mediated Cardiomyopathy and Potential Therapeutic Strategies. Biomolecules.

[248] Stojanovic B., Jovanovic I., Dimitrijevic Stojanovic M., Stojanovic B.S., Kovacevic V., Radosavljevic I., Jovanovic D., Miletic Kovacevic M., Zornic N., Arsic A.A. (2025). Oxidative Stress-Driven Cellular Senescence: Mechanistic Crosstalk and Therapeutic Horizons. Antioxidants.

[249] Nalobin D., Alipkina S., Gaidamaka A., Glukhov A., Khuchua Z. (2020). Telomeres and Telomerase in Heart Ontogenesis, Aging and Regeneration. Cells.

[250] Mahoney S.A., Bloom S.I., Seals D.R., Donato A.J., Rossman M.J., Clayton Z.S. (2025). Mechanisms of cellular senescence-induced vascular aging: Evidence of senotherapeutic strategies. J. Cardiovasc. Aging.

[251] Sandler N., Kaczmarek E., Itagaki K., Zheng Y., Otterbein L., Khabbaz K., Liu D., Senthilnathan V., Gruen R.L., Hauser C.J. (2018). Mitochondrial DAMPs Are Released During Cardiopulmonary Bypass Surgery and Are Associated with Postoperative Atrial Fibrillation. Heart Lung Circ..

[252] van Deursen J.M. (2019). Senolytic therapies for healthy longevity. Science.

[253] Vaduganathan M., Docherty K.F., Claggett B.L., Jhund P.S., de Boer R.A., Hernandez A.F., Inzucchi S.E., Kosiborod M.N., Lam C.S.P., Martinez F. (2022). SGLT-2 inhibitors in patients with heart failure: A comprehensive meta-analysis of five randomised controlled trials. Lancet.

[254] Katsuumi G., Shimizu I., Suda M., Yoshida Y., Furihata T., Joki Y., Hsiao C.L., Jiaqi L., Fujiki S., Abe M. (2024). SGLT2 inhibition eliminates senescent cells and alleviates pathological aging. Nat. Aging.

[255] Lopaschuk G.D., Verma S. (2020). Mechanisms of Cardiovascular Benefits of Sodium Glucose Co-Transporter 2 (SGLT2) Inhibitors: A State-of-the-Art Review. JACC Basic Transl. Sci..

[256] Schernthaner G., Brand K., Bailey C.J. (2022). Metformin and the heart: Update on mechanisms of cardiovascular protection with special reference to comorbid type 2 diabetes and heart failure. Metabolism.

[257] Partridge L., Fuentealba M., Kennedy B.K. (2020). The quest to slow ageing through drug discovery. Nat. Rev. Drug Discov..

[258] Zhang L., Pitcher L.E., Prahalad V., Niedernhofer L.J., Robbins P.D. (2023). Targeting cellular senescence with senotherapeutics: Senolytics and senomorphics. FEBS J..

[259] Li T., Li S., Ma K., Kong J. (2024). Application potential of senolytics in clinical treatment. Biogerontology.

[260] Dinkova-Kostova A.T., Copple I.M. (2023). Advances and challenges in therapeutic targeting of NRF2. Trends Pharmacol. Sci..

